# Oncolytic adenovirus encoding apolipoprotein A1 suppresses metastasis of triple-negative breast cancer in mice

**DOI:** 10.1186/s13046-024-03011-0

**Published:** 2024-04-03

**Authors:** Jie Dong, Lingkai Kong, Shiqun Wang, Mao Xia, Yenan Zhang, Jingyi Wu, Fuming Yang, Shuguang Zuo, Jiwu Wei

**Affiliations:** 1 State Key Laboratory of Pharmaceutical Biotechnology, Jiangsu Key Laboratory of Molecular Medicine, Medical School of Nanjing University, 22 Hankou Road, Nanjing, Jiangsu 210093 P.R. China; 2 The Cancer Hospital of the University of Chinese Academy of Sciences (Zhejiang Cancer Hospital), Institute of Basic Medicine and Cancer (IBMC), Chinese Academy of Sciences, Hangzhou, Zhejiang China; 3 Department of Laboratory Medicine, Nanjing Drum Tower Hospital, Affiliated Hospital of Medical School, Nanjing University, Nanjing, P.R. China; 4 Liuzhou Key Laboratory of Molecular Diagnosis, Guangxi Key Laboratory of Molecular Diagnosis and Application, Affiliated Liutie Central Hospital of Guangxi Medical University, Liuzhou, Guangxi China

**Keywords:** Cholesterol, Triple-negative breast cancer, Metastasis, Keratin 14, FOXO3a, Apolipoprotein A1, Oncolytic virus

## Abstract

**Background:**

Dysregulation of cholesterol metabolism is associated with the metastasis of triple-negative breast cancer (TNBC). Apolipoprotein A1 (ApoA1) is widely recognized for its pivotal role in regulating cholesterol efflux and maintaining cellular cholesterol homeostasis. However, further exploration is needed to determine whether it inhibits TNBC metastasis by affecting cholesterol metabolism. Additionally, it is necessary to investigate whether ApoA1-based oncolytic virus therapy can be used to treat TNBC.

**Methods:**

In vitro experiments and mouse breast cancer models were utilized to evaluate the molecular mechanism of ApoA1 in regulating cholesterol efflux and inhibiting breast cancer progression and metastasis. The gene encoding ApoA1 was inserted into the adenovirus genome to construct a recombinant adenovirus (ADV-ApoA1). Subsequently, the efficacy of ADV-ApoA1 in inhibiting the growth and metastasis of TNBC was evaluated in several mouse models, including orthotopic breast cancer, spontaneous breast cancer, and human xenografts. In addition, a comprehensive safety assessment of Syrian hamsters and rhesus monkeys injected with oncolytic adenovirus was conducted.

**Results:**

This study found that dysregulation of cholesterol homeostasis is critical for the progression and metastasis of TNBC. In a mouse orthotopic model of TNBC, a high-cholesterol diet promoted lung and liver metastasis, which was associated with keratin 14 (KRT14), a protein responsible for TNBC metastasis. Furthermore, studies have shown that ApoA1, a cholesterol reverse transporter, inhibits TNBC metastasis by regulating the cholesterol/IKBKB/FOXO3a/KRT14 axis. Moreover, ADV-ApoA1 was found to promote cholesterol efflux, inhibit tumor growth, reduce lung metastasis, and prolonged the survival of mice with TNBC. Importantly, high doses of ADV-ApoA1 administered intravenously and subcutaneously were well tolerated in rhesus monkeys and Syrian hamsters.

**Conclusions:**

This study provides a promising oncolytic virus treatment strategy for TNBC based on targeting dysregulated cholesterol metabolism. It also establishes a basis for subsequent clinical trials of ADV-ApoA1 in the treatment of TNBC.

**Supplementary Information:**

The online version contains supplementary material available at 10.1186/s13046-024-03011-0.

## Introduction

Triple-negative breast cancer (TNBC) has a high rate of distant metastasis, and effective therapies are lacking. Patients with TNBC have poor overall survival, demanding further investigation into the factors driving the initiation and the progression of the disease [1]. Epidemiological and large-scale gene sequencing studies have reported high heterogeneity within TNBC tumors and almost no common actionable targets [2]. With the prevalence of obesity, hyperglycemia, hyperlipidemia, and hyperinsulinemia, metabolic disturbances are commonly developed, resulting in an increased incidence of TNBC [3]. The potential metabolic abnormalities that promote the progression of TNBC include increased total cholesterol, circulating low-density lipoprotein (LDL) cholesterol, triglycerides (TGs), insulin, and decreased high-density lipoprotein (HDL) cholesterol [4]. Elevated cholesterol biosynthesis is a hallmark of various cancers, including glioblastoma, colorectal cancer, and breast cancer [5, 6]. Previous studies showed that cholesterol might participate in tumor progression through at least three ways: (i) as initial substrates for bile acids, steroid hormones, and other cholesterol derivatives, which are pivotal risk factors for cancers [7, 8]; (ii) covalently modulating proteins involved in tumorigenesis and progression, such as hedgehog and smoothened [9]; (iii) assisting the function of lipid raft to regulate the signal receptors required for tumorigenesis [10]. In addition, cholesterol metabolism is also critical for sustaining the activities of immune cells including effector T cells, regulatory cells (Tregs), T Helper 17 (Th17) cells, and macrophages, and therefore determines antitumor immune responses [11]. Nevertheless, the mechanisms by which aberrant cholesterol metabolism promotes TNBC progression and metastasis are not yet fully understood.

Previous studies have consistently demonstrated a strong correlation between high expression of LDLR, a receptor responsible for the uptake of exogenous cholesterol, and lower overall and recurrence-free survival in patients with breast cancer [12]. Given the crucial role of cholesterol in cancer progression, manipulating abnormal cholesterol metabolism has emerged as a potential therapeutic strategy, including inhibiting the de novo synthesis pathway, suppressing cholesterol esterification, and activating the LXR signal pathway [13, 14]. Several clinical studies have reported the beneficial effects of cholesterol reduction, achieved through the use of statins, on the survival of patients with various cancer types [15–17]. However, the impact of cholesterol-lowering specifically in TNBC remains inconclusive [5, 18, 19].

Apolipoprotein A1 (ApoA1), the predominant protein component of HDL, is widely recognized for its pivotal role in regulating cholesterol transportation and maintaining cellular cholesterol homeostasis [20]. Although most studies have focused on the role of ApoA1 in atherosclerosis and cardiovascular disease, some studies have revealed relatively lower levels of serum ApoA1 in patients with various cancers, including breast cancer [21–23]. Particularly, patients with TNBC have shown significantly decreased serum ApoA1 levels compared to luminal A and B molecular subtypes [24]. Furthermore, a postoperative serum proteomics analysis conducted on high-risk breast cancer patients revealed a correlation between low ApoA1 expression and the occurrence of metastatic relapse [25]. Nevertheless, the precise mechanisms underlying the impact of ApoA1 on TNBC progression and metastasis remain incompletely understood.

Oncolytic viruses (OVs) are viruses that occur naturally or are genetically engineered to selectively replicate within tumor cells, leading to their destruction while sparing normal cells [26]. Adenovirus, herpes simplex virus, and vaccinia virus are currently the three most commonly utilized viruses in preclinical studies and clinical trials for cancer treatment [27]. Adenoviruses are one of the most widely studied viruses due to their several advantages, such as ease of genome manipulation and the feasibility of manufacturing high viral titers. In our previous study, the intratumoral injection of ADV-ApoA1 was shown to reprogram the lipid metabolism of glioblastoma multiforme (GBM), restore TAMs’ phagocytosis, and reactivate T cell anti-tumor immunity. However, it remains unclear whether ADV-ApoA1 can exert similar antitumor effects in breast cancer.

In the present study, we aimed to investigate the impact of cholesterol on TNBC metastasis and explore potential therapeutic approaches by modulating cholesterol metabolism. Our findings revealed that a high-cholesterol diet promotes TNBC metastasis by upregulating the expression of KRT14 in a mouse model. Conversely, we observed that ApoA1 inhibits TNBC metastasis by regulating the cholesterol/IKBKB/FOXO3a/KRT14 axis. Building upon these discoveries, we developed an oncolytic adenovirus encoding ApoA1 and assessed its efficacy in suppressing TNBC growth and metastasis across diverse mouse models, including orthotopic breast cancer, spontaneous breast cancer, and human xenograft transplants. Furthermore, we conducted a comprehensive safety assessment of the oncolytic adenovirus in Syrian hamsters and rhesus monkeys.

## Materials and methods

### Cell lines

The cell lines employed in this study were obtained from the American Type Culture Collection (ATCC, USA), including 293T cells (human embryonic kidney cells), HCC1937 cells, MDA-MB-231 cells (human triple negative-breast carcinoma), and 4T1 cells (mouse mammary carcinoma cells). These cell lines were cultured in Dulbecco’s modified Eagle’s medium (DMEM, Gibco-Thermo Fisher Scientific, USA) supplemented with 10% fetal bovine serum (FBS, Gibco). Specifically, 293T cells were cultured in suspension using a serum-free medium (Basalmedia, Shanghai, China) in spinner flasks (Jetbiofil, Guangzhou, China). To generate the 4T1^ApoA1^ and HCC1937^ApoA1^ cells, lentiviral transduction was employed followed by subsequent puromycin selection. All cell lines were incubated at 37 °C in a 5% CO_2_ atmosphere.

### CRISPR screening in 4T1 cells in vivo

The Genome-scale CRISPR-Cas9 transcriptional activation screening method employed in this study has been previously described [28, 29]. In brief, lentiviruses for establishing the transcription activation system were obtained from Genechem Company (Shanghai, China). Cells were plated at a low density of 1 × 10^7^ cells per T225 Flask, and the Lenti-sgRNA library was added at a MOI of 0.5. After 48 h, cells were selected in the presence of 2 µg/ml puromycin. The in vivo genome-scale screening was conducted following a previously published protocol [30]. To deplete CD8^+^ T cells, mice were injected with CD8 antibodies one day before tumor inoculation. One day later, BALB/c mice (*n* = 6) were injected subcutaneously with 4T1 cells (5 × 10^6^) containing the transcription activation system. On days 5 and 10 after tumor inoculation, mice were sacrificed (*n* = 3 for each time point), and tumors were collected for genomic DNA extraction. Significantly enriched or depleted sgRNAs were identified by comparing different time points, as described in a previous report [31].

### Online bioinformatic analysis

The correlation between cholesterol biosynthesis/efflux genes and the survival of breast cancer patients was analyzed using the Gene Expression Profiling Interactive Analysis (GEPIA) online bioinformatic tool (http://gepia.cancer-pku.cn) base on the tumor transcriptome sequencing data of breast cancer patients from The Cancer Genome Atlas Program (TCGA).

### Cholesterol efflux assay

Cholesterol efflux was measured using a cholesterol efflux assay kit (Biovision, CA, USA). Cells were seeded in a 96-well tissue culture plate with 100 µl of growth medium per well. A labeling medium was prepared by mixing Labeling Reagent and serum-free RPMI medium. Cells were then incubated in the labeling medium for 1 h. Subsequently, the medium was replaced with an equilibration medium. The medium was aspirated, and 2% LDL/VLDL-depleted cholesterol acceptors, diluted in phenol red-free, serum-free RPMI medium, were added. The plate was incubated for another 4 h, after which the fluorescence intensity (RUF) of the supernatant and cell lysis was measured (Ex/Em = 485/523 nm). Cholesterol efflux was calculated using the formula: Cholesterol efflux= [RUF(supernatants)/ RUF(supernatants + cells)] ×100%. The absolute concentration of intracellular cholesterol was measured using spectrophotometric tests (Nanjing Jiancheng, Nanjing, China).

### Chromatin immunoprecipitation sequencing (ChIP-Seq) analysis

For the ChIP-Seq experiment, we utilized the Rabbit anti-FOXO3a antibody (#ab12162) obtained from Abcam, along with the Enzymatic Chromatin IP kit from Cell Signaling Technology. To begin, 4T1^ApoA1^ cells were cultured to reach 80% confluency and then cross-linked using 1% formaldehyde at room temperature for 10 min. To prepare the ChIP DNA, chromatin was digested with micrococcal nuclease and subsequently sonicated to generate fragments ranging in size from 100 to 500 base pairs. Following this, RNAse A and proteinase K were employed for further digestion, and the ChIP DNA was incubated overnight with 4 µg of the anti-FOXO3a antibody. The ChIP DNA was then purified using a column system based on magnetic separation, and the resulting eluted DNA was used as the input for the subsequent Chip-seq analysis. The DNA sequencing and analysis procedures were carried out by Novogene (Beijing, China).

### Wound healing assays

Cells were seeded at a density of 5 × 10^4^ cells per well in 12-well plate and incubated in DMEM without FBS. Once the cells reached 80% confluence, a scratch was created across the surface of each well using a 10-µl pipette. The scratches were then observed at specified time points after the incubation period.

### Transwell assays

Transwell assays were performed using 8 μm pore size Transwell chambers (Corning, NY, USA) that were pre-coated with rat tail type I collagen (Corning, NY, USA). The cells were cultured in the Transwell chambers for 24 h. Following the incubation period, the cells present on the upper surface of the chamber membrane were carefully eliminated using cotton swabs, while the cells on the lower surface of the membrane were subjected to staining using a solution composed of methanol and 0.1% crystal violet. The invaded cells were subsequently observed and captured using a microscope (Olympus, Tokyo, Japan). For each chamber, five random visual fields were selected for analysis, with a 100 × magnification.

### Three-dimensional matrigel culture

For Three-dimensional matrigel culture, a droplet of 20 µl medium containing 100 cells was placed on the lower surface of the dish cover and incubated overnight. Subsequently, the droplets were seeded onto the Matrigel gel (Corning, NY, USA). After a designated growth period, images were captured using an inverted microscope at a magnification of 100 ×.

### Quantitative real-time polymerase chain reaction (qRT-PCR)

Total RNA was extracted using Trizol (Invitrogen) following the manufacturer’s instructions. Reverse transcription was performed, and qPCR was carried out using the QuantStudio 5 instrument (Applied Biosystems). GAPDH was used as an internal reference and co-amplified with the target samples under identical qPCR conditions. The primer sequences for qPCR are listed in Table S1.

### siRNA transfection

siRNAs were obtained from GenePharm (Shanghai, China). Cells were transfected with siRNA (100 nM) for 48 or 72 h using Lipofectamine RNAi MAX Reagent (ThermoFisher Scientific, MA, USA). The specific siRNAs used are provided in Table S2.

### Western blot analysis

Cell culture medium was removed from the dishes, and the cells were lysed in RIPA lysis buffer (Cell Signaling Technology, MA, USA) supplemented with a protease inhibitor cocktail (ThermoFisher Scientific). The supernatants from cell lysis were collected and mixed with a loading buffer containing 2-mercaptoethanol (2-ME), followed by heating at 95 °C for 5 min. After separation on a 10% SDS-PAGE gel, the proteins were transferred to a polyvinylidene fluoride (PVDF) membrane (Merck Millipore, MA, USA) and incubated with specific primary antibodies including rabbit anti-KRT14 antibody (Abcam Cambridge, UK), rabbit anti-FOXO3a antibody, anti-GAPDH, anti-β-actin, anti-phospho-Akt, anti-phospho-Ikb (Cell Signaling Technology), rabbit anti-histone H3.1 (Abmart, Shanghai, China), and mouse anti-Flag (GenScript Biotech, Nanjing, China). After washing, the membrane was probed with horseradish peroxidase (HRP)-conjugated anti-mouse or rabbit IgG (GenScript) and visualized using a western blot chemiluminescence reagent (GE Healthcare, Giles, UK) and an imaging system (Sage Creation Science, Beijing, China).

### Immunofluorescence

For immunofluorescence staining, cells cultured on poly-L-Lysine-coated coverslips were fixed with 4% PFA and permeabilized using 0.25% Triton-X. The cells were then incubated overnight at 4 °C with the primary antibody, rabbit anti-FOXO3a (Cell Signaling Technology), followed by incubation with an AlexaFluor568-conjugated secondary antibody. Nuclei were counterstained with Hoechst 33,258 (Beyotime Biotechnology, Shanghai, China). The rabbit anti-KRT14 antibody (Abcam) was used as the primary antibody for the frozen section of the tumor tissue.

### Cholesterol deprivation and replenishment

To induce cholesterol deprivation, cells were treated with 5 mmol/L LXR623 (MedChemExpress, Monmouth Junction, NJ, USA) for 48 h. For cholesterol replenishment, cholesterol-saturated Methyl-β-cyclodextrin (MβCD-cholesterol) was prepared. In brief, cholesterol was dissolved in chloroform: methanol (1:1 vol/vol) and added to a glass tube for solvent evaporation. The dried cholesterol was then mixed with a DMEM containing 5 mM MβCD. The sealed tube was incubated overnight in a shaking bath at 37 ℃.

### Recombinant oncolytic adenovirus construction, purification, and expansion

The shuttle plasmid for recombinant adenovirus construction was synthesized by GenScript (Nanjing, China). This plasmid contained the reporter gene EGFP and adenovirus E1A gene, which were linked by a T2A peptide sequence and controlled by the CMV promoter. The expression of ApoA1 was governed by the EF1a promoter. The recombinant oncolytic adenovirus expressing ApoA1 was obtained through homologous recombination between the shuttle plasmid and the adenovirus backbone pAd/PL-DEST (Thermo Fisher Scientific, Invitrogen, Carlsbad, CA, USA). After digestion with the restriction enzyme PacI, the linear recombinant adenoviral vector was transfected into 293T cells using the jet-PEI transfection reagent (Cat# 101-10 N, Polyplus-transfection, France). The recombinant adenovirus was then amplified in 293T cells and purified via iodixanol gradient ultracentrifugation. Virus titration was determined by adding serially diluted virus to a 96-well plate seeded with 293T cells (10,000 cells/well). The cells were cultured for 4 days, and fluorescence plaques were counted. The virus titer was calculated using the following formula: TCID50 = 10^2^ + (S/10 − 0.5) / ml, pfu/ml = 0.7 × TCID50/ml, where S is the total number of fluorescence-positive wells and N is the number of replicates.

### Replication of the oncolytic adenovirus in tumor cells

4T1 cells were seeded at a density of 5 × 10^4^ cells per well in a 24-well plate and incubated in a 5% CO_2_ incubator at 37 °C. Once the cells reached 90% confluence, they were infected with the recombinant oncolytic adenovirus at a multiplicity of infection (MOI) of 0.1. After 24, 48, 72, and 96 h, the cells were harvested, subjected to three freeze-thaw cycles, and centrifuged at 3000 × g for 10 min. The virus supernatants were collected for viral genome extraction. The number of virus copies was determined using qPCR.

### Animal models

All animal experiments were conducted according to the guidelines approved by the Animal Care and Use Committee of the Medical School of Nanjing University. Six-week-old female BALB/c mice, nude mice, and NCG (NOD/ShiLtJGpt-Prkdc^em26Cd52^Il2rg^em26Cd22^/Gpt) mice were purchased from GemPharmatech Co., Ltd. (Nanjing, China). Female MMTV-PyMT mice were purchased from Nanjingziyuan Co., Ltd (Nanjing, China).

To establish an orthotopic tumor model, BALB/c mice were orthotopically inoculated with 4T1 or 4T1-luciferase cells into the fourth mammary glands. To establish a humanized MDA-MB-231 model, NCG mice were orthotopically inoculated with MDA-MB-231 cells into the fourth mammary glands. When the tumor volume reached 100 mm^3^, mice received intravenous injection of 2 × 10^6^ human peripheral blood mononuclear cells (PBMCs). Mice were sacrificed if the tumor volume exceeded 2000 mm^3^. Tumor length (L) and width (W) were measured every two days using Vernier calipers, and the tumor size (V) was calculated using the formula: V = (L × W^2^)/2.

For high-lipid feeding, tumor-bearing BALB/c mice were fed a Western or high-cholesterol diet. The Western diet contained 1.25% cholesterol and 0.5% sodium cholate, while the high-cholesterol diet contained 1.5% cholesterol and 0.5% sodium cholate. These diets provided approximately 40% kilocalories from fat, 20% from protein, and 40% from carbohydrates for the Western diet, and approximately 9.4% kilocalories from fat, 14.7% from protein, and 75.9% from carbohydrates for the high-cholesterol diet. The diets were obtained from Dytes Inc. (Wuxi, Jiangsu, China).

For hydrodynamic administration, the orthotopic model (luciferase) was established as previously described. Ten days later, the primary tumor was surgically excised, and the ApoA1 expression plasmid (10 µg/mouse) was injected via the tail vein once a week. Lung metastasis was monitored by bioluminescence analysis on day 40.

For oncolytic virus therapy, upon reaching a tumor size of 100 mm^3^ (day 0), mice were randomly assigned to various groups and administered intratumoral (I.T.) injections of saline, ADV-ApoA1, or ADV-Ctr (5 × 10^8^ PFU) on days 1, 3, and 5. MMTV-PyMT mice with spontaneous breast cancer underwent intratumoral injections of ADV-ApoA1 (5 × 10^8^ PFU) every week for two weeks. The presence of lung metastasis was assessed three weeks later.

### Single-sample gene set Enrichment Analysis (ssGSEA)

The ssGSEA was used to quantify immune cell infiltration, which was performed via R packages (GSVA, version 3.18) as previous describe [32].

### H&E staining and immunohistochemistry (IHC)

The tumor tissues were fixed with 4% paraformaldehyde and subsequently sectioned into 5-µm-thick slides. These slides were then subjected to standard H&E staining procedures. For IHC analysis, the slides were incubated overnight at 4 °C with a primary antibody against phosphorylated p65 (Cell Signaling Technology, Danvers, MA, USA). Following this, the slides were incubated with HRP-conjugated streptavidin for 1 h at room temperature. Visualization was achieved using the DAB chromogen (Zhongshan Golden Bridge, Beijing, China). Images were captured at 100 × and 400 × magnifications using a microscope (Zeiss, Oberkochen, Germany).

### Toxicity of ADV-ApoA1 in Syrian hamster and Rhesus monkey

A total of 30 Syrian hamsters were randomly assigned to 3 groups based on body weight, with each group consisting of 5 male and 5 female hamsters in equal proportions. In the dose range finding (DRF) groups, the hamsters were intraperitoneally (I.H.) or intravenously (I.V.) administered either 5 × 10^12^ VP/kg or 1.2 × 10^12^ VP/kg of the ADV-ApoA1 for five consecutive days. After a two-day rest period, the hamsters were treated with the virus for another five consecutive days. A negative control was used, consisting of a solvent without viral particles. Blood samples were collected 24 h after the final viral injection for clinical chemistry tests.

The same procedures were performed on Rhesus monkeys (*n* = 10) for DRF. For MTD evaluations, the Rhesus monkeys (*n* = 4) received a single treatment of 2 × 10^13^ VP/kg of ADV-ApoA1 either intraperitoneally (I.H.) or intravenously (I.V.). For Histological examination, Rhesus monkeys were subcutaneous injected with ADV-ApoA1 (1.5 × 10^12^ VP/kg) for 6 times. Tissues were collected 24 h after the last viral injection.

### Statistical analysis

The statistical analyses were conducted using GraphPad Prism software (version 9.5.1, La Jolla, California, USA). The data were presented as mean ± SD. A Student t-test or One-way ANOVA analysis was employed to determine the differences between groups. Correlation analysis was performed using Pearson or Spearman methods. A significance level of *P* < 0.05 was considered statistically significant.

## Results

### Dysregulation of cholesterol metabolism promotes the progression and metastasis of TNBC

**Fig. 1:**
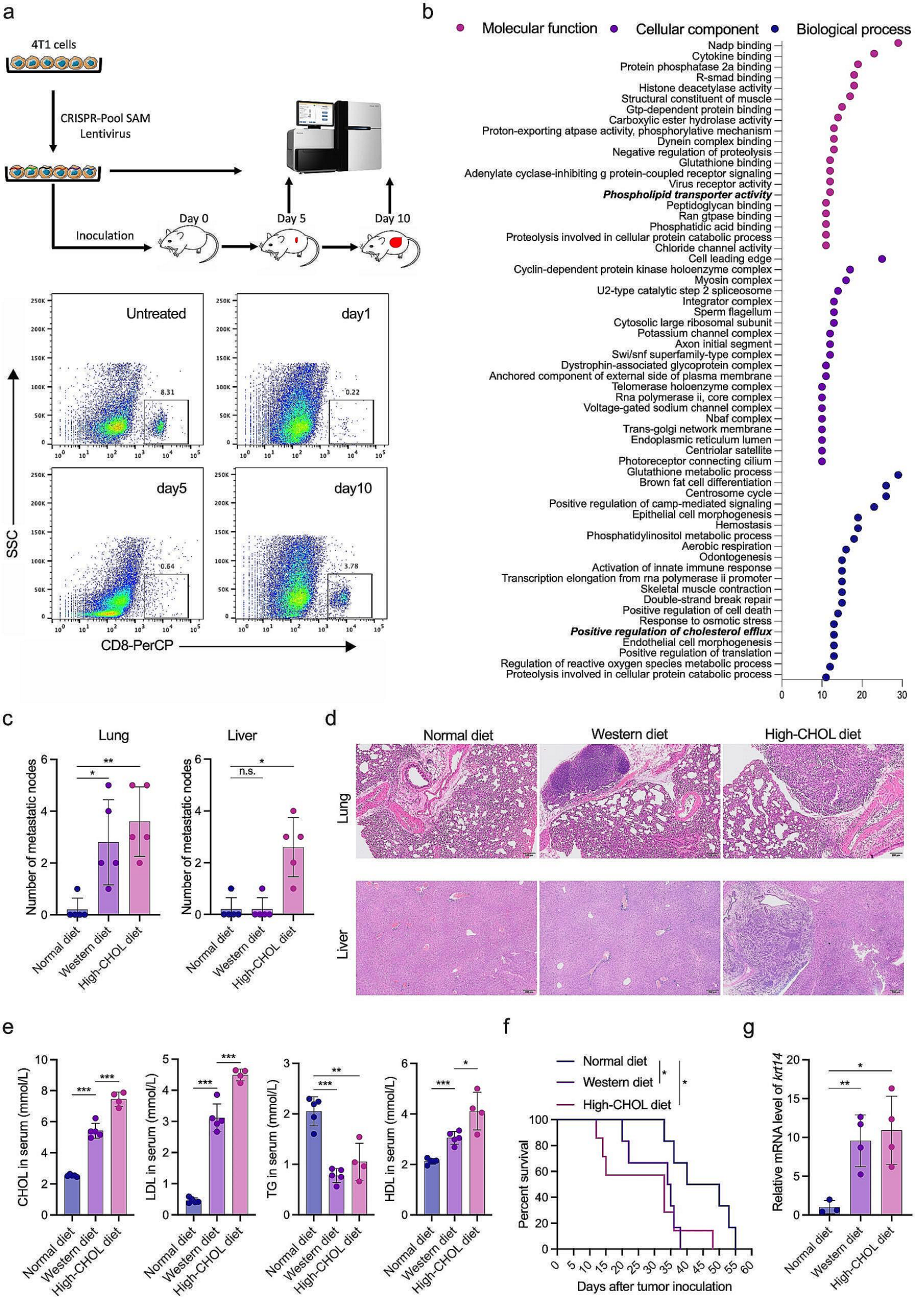
Cholesterol metabolism was pivotal for TNBC progression. (a) Schematic representation of the in vivo screen using the mouse genome-scale CRISPR-Pool SAM library. 4T1 cells were infected with lentiviral CRISPR-Pool SAM library, and subcutaneously (s.c.) inoculated into mice. CD8^+^ T cells were depleted by injecting CD8 antibodies one day prior to tumor inoculation to allow tumor formation. Tumors were collected on days 5 and day 10 for sgRNA sequencing. (b) Bubble graph depicting the Gene Ontology (GO) enrichment analysis of gene sets in tumor tissues between day 5 and day 10. (c) 4T1 cells were s.c. inoculated into BALB/c mice and fed with a western diet, high-cholesterol diet, or normal diet. Mice were sacrificed on day 14 (*n* = 5) and the metastatic nodes were counted. (d) Representative images of H&E staining of lung and liver tissues from mice. (e) Serum was collected on day 10 and cholesterol (CHOL), low-density lipoprotein (LDL), high-density lipoprotein (HDL) and Triglyceride (TG) levels were measured. (f) Survival of mice receiving different diets was monitored. (g) Relative mRNA levels of krt14 in tumor tissues were measured using qPCR. *p < 0.05; **p < 0.01; ***p < 0.001

To explore the essential metabolic pathways involved in TNBC progression, we conducted a CRISPR/Cas9 gain-of-function screen using a pooled lentiviral synergistic activation mediator (SAM) library. The 4T1 cells were infected with the library [29] and then transplanted into CD8^+^ T-cell-depleted mice to facilitate tumor growth [33]. Tumors were collected on days 5 and 10 to assess tumor formation and progression, respectively (Fig. 1a). Comparing the representation of the sgRNA library, we observed significant implications of pathways associated with phospholipid transporters and cholesterol efflux in the progression of 4T1 breast cancer (Fig. 1b). Gene expression profiles of breast cancer and normal tissue from TCGA and The Genotype-Tissue Expression (GTEx) project indicated that gene sets related to cholesterol transport and efflux were enriched in breast cancer, while gene sets associated with cholesterol biosynthesis were downregulated in breast cancer (Fig. S1a). Moreover, gene sets for cholesterol transport and efflux were more enriched in TNBC than other molecular types (nTNBC) (Fig. S1b).

To further investigate the correlation between the dysregulation of cholesterol metabolism and the progression of TNBC, we established a 4T1 orthotopic breast cancer xenograft model and fed tumor-bearing mice a high-cholesterol diet to examine the impact of cholesterol on tumor metastasis. In this model, the consumption of a high-cholesterol diet resulted in a notable increase in the number of metastatic nodules in both the lung and liver, whereas a Western diet (high fat and cholesterol) solely amplified the number of metastatic nodules in the lungs (Fig. 1c, d). Compared to mice on a normal diet, those fed a Western diet or a high-cholesterol diet exhibited an approximately 2- to 3-fold increase in serum cholesterol levels, a 6- to 9-fold increase in serum LDL levels, and a 1.5 to 2-fold increase in serum HDL levels. Conversely, TG levels were observed to decrease by half in mice on these diets (Fig. 1e). Furthermore, both the Western diet and the high-cholesterol diet significantly shortened the survival time of tumor-bearing mice (Fig. 1f). Additionally, we observed an elevated expression of KRT14 in tumor tissue from the mice fed either a Western diet or a high-cholesterol diet (Fig. 1g). Further analysis revealed that breast cancer patients with higher expression levels of cholesterol efflux-related genes, such as ApoA1 and NR1H3 (also known as LXRa), appeared to have extended survival rates. Surprisingly, the expression of genes SREBF1, SREBF2, or HMGCR, which are key regulators of cholesterol biosynthesis, did not impact survival outcomes (Fig. S1c). These findings strongly suggest that dysregulation in cholesterol metabolism plays a pivotal role in the progression and metastasis of TNBC.

### ApoA1 suppresses the lung metastasis of TNBC by promoting cholesterol efflux

**Fig. 2:**
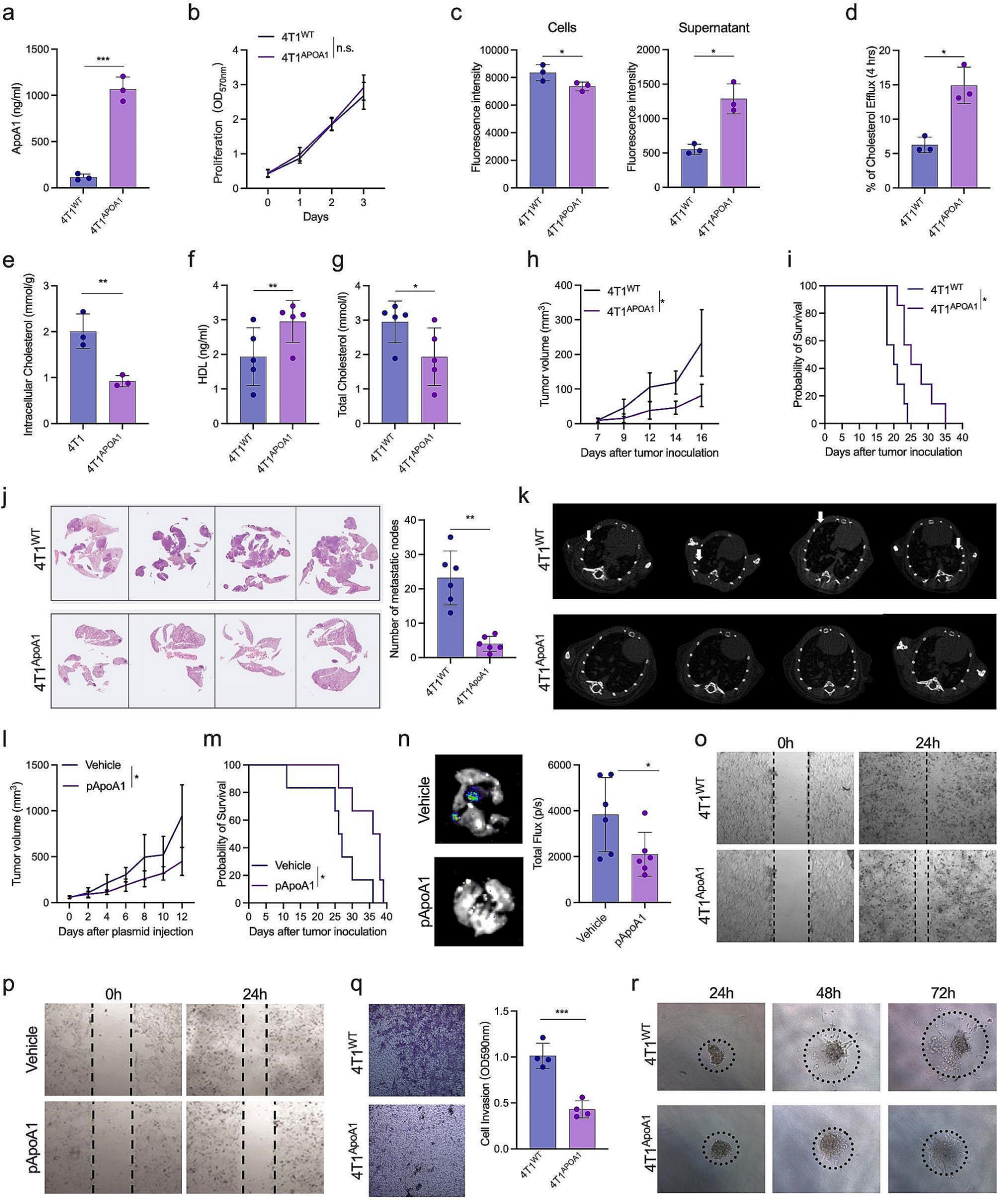
ApoA1 inhibited metastasis of TNBC. (a) A stable 4T1 cell line overexpressing ApoA1, 4T1ApoA1, was generated through lentivirus infection, and the expression of ApoA1 was detected using ELISA. (b) Proliferation of 4T1^ApoA1^ and the parental cell line 4T1^WT^ was determined using MTT assay. (c) and (d) Cholesterol efflux was measured using a cholesterol efflux assay kit. (e) Intracellular cholesterol levels were measured using spectrophotometric tests. BALB/c mice were orthotopically inoculated with 4T1^WT^ and 4T1^ApoA1^ cells. (f) and (g) Serum was collected on day 10 to measure high-density lipoprotein (HDL) and total cholesterol (TC) levels. (h) Tumor volume in the orthotopic mouse model. (i) Survival of tumor-bearing mice (*n* = 7). (j) Mice were sacrificed on day 16 (*n* = 5) to calculate lung metastasis, and representative H&E staining images are shown. k) Micro-CT image of lung metastasis. l) and m) Tumor volume of the 4T1-luciferase orthotopic mouse model and survival of mice (*n* = 7). Mice were high-pressure hydrodynamically injected with an ApoA1 expression plasmid (pApoA1) via the tail vein. n) Lung metastasis of the 4T1-luciferase orthotopic mouse model, in which the primary tumor was surgically excised 10 days after tumor inoculation. o) Wound healing assays of 4T1^WT^ or 4T1^ApoA1^ cells. p) Migration of 4T1 cells transfected with either ApoA1 expressing plasmid (pApoA1) or control plasmid (vehicle) was tested using wound healing assays. q) Invasion of 4T1^WT^ or 4T1^ApoA1^ was evaluated by Transwell assays. Invasive cells were stained with crystal violet (left), and absorbance was measured at 590 nm after reconstitution of crystal violet (right). r) Colony formation of 4T1^WT^ or 4T1^ApoA1^ cells cultured in Matrigel and imaged at 24, 48, and 72 h. *p < 0.05; **p < 0.01; ***p < 0.001

ApoA1, a major constituent of HDL, has been demonstrated to facilitate cholesterol efflux and reverse transport [34]. To investigate whether ApoA1 could influence the progression and metastasis of TNBC by promoting cholesterol efflux, we employed lentiviral vectors to transduce 4T1 and MDA-MB-231 cells, resulting in the generation of stable cell lines overexpressing ApoA1, namely 4T1^ApoA1^ and MDA-MB-231^ApoA1^. As expected, ApoA1 was efficiently produced and secreted into the culture medium of 4T1^ApoA1^ cells (Fig. 2a). Furthermore, we observed that the transgene of ApoA1 did not alter the proliferation characteristics of 4T1^ApoA1^ and MDA-MB-231^ApoA1^ cells (Fig. 2b; Fig. S2a), but significantly enhanced cholesterol efflux in these cells, leading to a notable reduction in intracellular cholesterol levels. (Fig. 2c-e; Fig. S2b-d). In comparison to mice bearing 4T1^WT^ tumors, mice bearing 4T1^ApoA1^ tumors exhibited elevated levels of HDL in their sera, along with decreased levels of cholesterol (Fig. 2f, g). Furthermore, mice bearing 4T1^ApoA1^ tumors displayed decelerated tumor growth, and prolonged survival compared to mice bearing 4T1^WT^ tumors (Fig. 2h, i). Histological examination and micro-computed tomography (CT) imaging further revealed that the prolonged survival of 4T1^ApoA1^-bearing mice was attributed to a reduction in lung metastatic nodules (Fig. 2j, k). Similarly, hydrodynamic administration of ApoA1-expressing plasmids through the tail veins of mice significantly impeded the growth of 4T1 orthotopic xenografts and significantly prolonged the survival of tumor-bearing mice. (Fig. 2l-m). Moreover, the administration of the ApoA1-expressing plasmid significantly decreased lung metastases in 4T1 orthotopically implanted mice following primary tumor resection (Fig. 2n). Consistent with these findings, impaired migration and invasion were observed in ApoA1-overexpressing TNBC cells, as evidenced by wound-healing and Transwell chamber assays (Fig. 2o-q; Fig. S2e). Additionally, the results of the three-dimensional culture demonstrated that 4T1^ApoA1^ cells exhibited weaker clonogenic and invasive abilities compared to their parental 4T1 cells. (Fig. 2r). These results indicated that ApoA1 has the potential to suppress lung metastasis in TNBC by promoting cholesterol efflux.

### ApoA1 inhibits lung metastasis of TNBC by downregulating the expression of KRT14

**Fig. 3:**
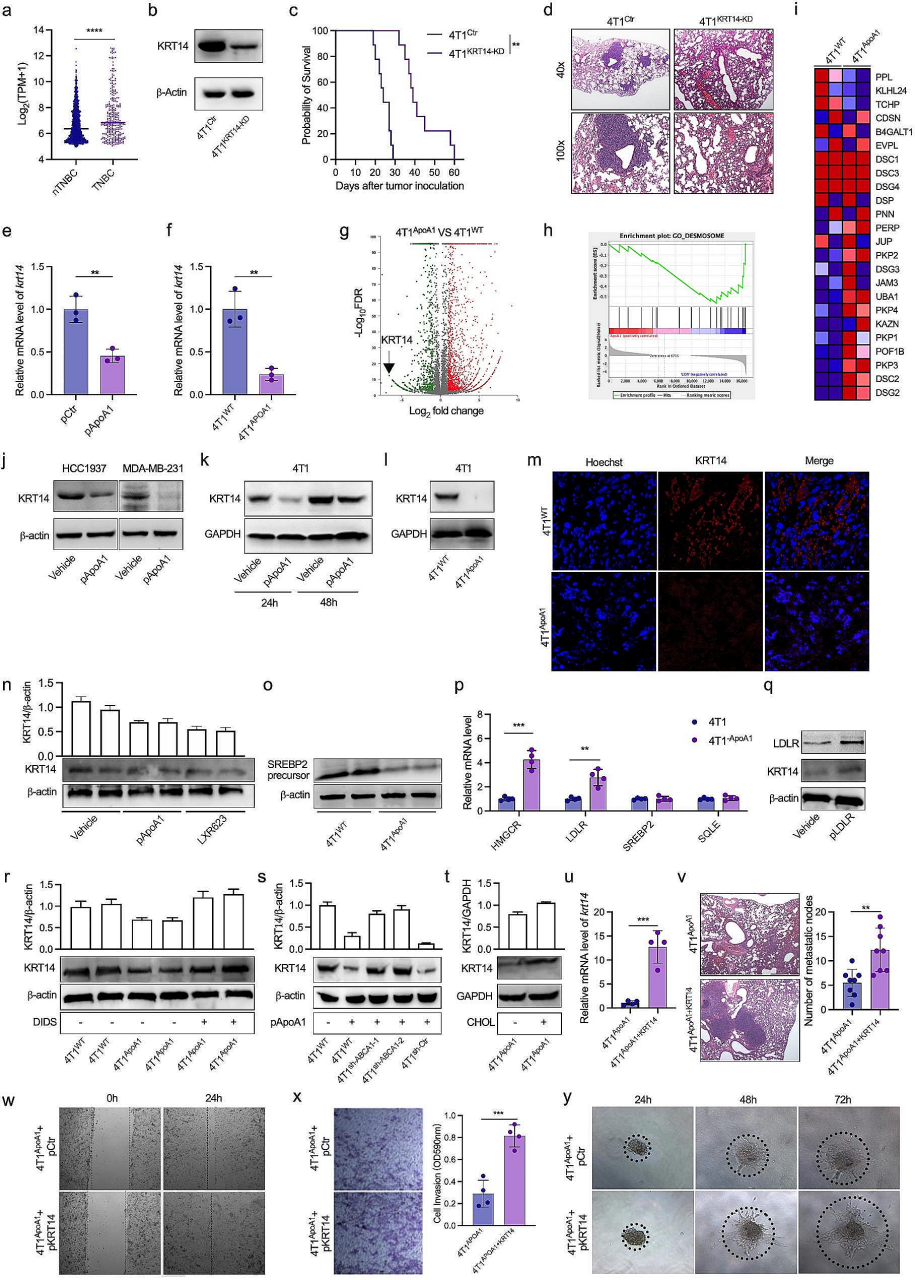
ApoA1 inhibit metastasis of TNBC trough downregulating KRT14. (a) KRT14 expression in tumor samples from patients with TNBC (*n* = 299) and other types of breast cancers (non-TNBC, *n* = 1605). Data collected from TCGA. (b) The KRT14 knockdown cell line, 4T1^KRT14 − KD^, was generated using lentivirus containing KRT14-specific shRNA, and KRT14 expression was confirmed by western blotting. (c) and (d) Survival curve and lung metastasis of mice orthotopically inoculated with 4T1^KRT14 − KD^ or 4T1^Ctr^. (e) and (f) mRNA expression of KRT14 in 4T1^ApoA1^ and 4T1 cells infected with pApoA1 plasmid, tested using qPCR. (g) Volcano plots of transcriptome comparison between 4T1^ApoA1^ and 4T1^WT^ cells. (h) and (i) GSEA and expression pattern of desmosome-associated genes in 4T1^WT^ and 4T1^ApoA1^ cells. (j) and k) Expression of KRT14 in 4T1, MDA-MB-231, and HCC1937 cells transfected with pApoA1 plasmid, tested using western blotting. l) Expression of KRT14 in 4T1^ApoA1^ cells. m) Orthotopically injected 4T1WT and 4T1ApoA1 cells into BALB/c mice. Tumors were collected on day 7 for immunofluorescence analysis of KRT14. n) Expression of KRT14 in 4T1 cells in the presence of LXR623. o) Expression of the precursor of sterol regulatory element-binding transcription factor 2 (SREBP2, 125 kDa) in 4T1^ApoA1^ and 4T1^WT^ cells. p) mRNA levels of cholesterol uptake or de novo synthesis-related genes. HMGCR: 3-hydroxy-3-methylglutaryl-CoA reductase; LDLR: low-density lipoprotein receptor; SQLE: squalene epoxidase. q) Expression of LDLR and KRT14 in 4T1 cells transfected with LDLR-encoding plasmid pLDLR. r) 4T1^ApoA1^ cells were treated with or without 1mM of DIDS, and KRT14 expression was determined 24 h later using western blotting. s) Expression of KRT14 in ABCA1 knockdown 4T1 cells (4T1sh-ABCA1) transfected with pApoA1 plasmid. Cells were collected 48 h after pApoA1 transfection. t) Expression of KRT14 in 4T1^ApoA1^ cells in the presence of 2 μm of cholesterol-saturated methyl-b-cyclodextrin. u) mRNA expression of KRT14 in 4T1^ApoA1 + KRT14^ cells generated using a KRT14-encoding lentivirus. v) Lung metastasis of mice orthotopically inoculated with 4T1^ApoA1 + KRT14^ or 4T1^ApoA1^. w-y) The migration, invasion, and colony formation abilities of 4T1^ApoA1^ cells transfected with either KRT14 expressing plasmid (pKRT14) or control plasmid (pCtr). w) Cell mgration was tested using wound healing assays. x) Cell invasion was evaluated using transwell assays and stained with crystal violet. y) Colony formation of cells was analyzed using colony formation assays in Matrigel and imaged at 24, 48, and 72 h. **p < 0.01; ***p < 0.001; ****p < 0.0001

KRT14 is a structural component of the cytoskeleton and is known to be positively expressed in the leader tumor cell clusters that collectively disseminate in breast cancer metastasis [35]. Notably, distant metastases appear to occur more frequently in TNBC patients compared to non-TNBC patients. Consistent with these observations, our analysis of the TCGA dataset confirmed that the expression of KRT14 in tumor samples from TNBC patients was higher than in samples from non-TNBC patients (Fig. 3a). In vivo experiments further validated the role of KRT14 in metastasis, as mice challenged with KRT14-knockdown 4T1 cells (4T1^KRT14 − KD^) exhibited a longer lifespan and reduced lung metastasis compared to mice challenged with control 4T1 cells (4T1^Ctr^) (Fig. 3b-d). Next, to explore the regulatory effect of ApoA1 on KRT14 expression at the transcriptional level, we performed qRT-PCR assays. The results demonstrated a significant decrease in KRT14 mRNA levels in both ApoA1-overexpressing plasmid-transfected 4T1 cells or the 4T1^ApoA1^ cells (Fig. 3e, f). Consistently, transcriptome analysis revealed a prominent downregulation of KRT14 in 4T1^ApoA1^ cells compared to 4T1^WT^ cells (Fig. 3g). Gene set enrichment analysis (GSEA) of differentially expressed genes further supported these findings, showing higher mRNA levels of desmosome-related genes in 4T1^WT^ cells compared to 4T1^ApoA1^ cells (Fig. 3h, i), which is in line with previous reports linking KRT14 expression to desmosome adhesion complex genes [35]. At the protein level, transfection of ApoA1-overexpressing plasmid in HCC1937, MDA-MB231, and 4T1 cells resulted in a significant reduction in KRT14 expression (Fig. 3j, k). Consistently, reduced KRT14 expression was also observed in 4T1^ApoA1^ cells and tumor tissues obtained from orthotopic models established with these cells (Fig. 3l, m).

Given our previous findings on ApoA1-induced cholesterol efflux, we sought to explore whether the regulation of KRT14 expression by ApoA1 is linked to its effect on cholesterol efflux. To address this, we compared the effects of LXR623, an LXR agonist known to mediate cholesterol efflux, with ApoA1 on KRT14 expression. Similar to ApoA1, LXR623 was found to downregulate KRT14, suggesting that ApoA1-mediated cholesterol efflux may be involved in the suppression of KRT14 expression (Fig. 3n). Intracellular cholesterol efflux is often accompanied by upregulation of exogenous cholesterol uptake and *de novo* synthesis, primarily mediated by the pivotal modulator SREBP2. In line with this, we observed elevated proteolytic cleavage of SREBP2 in 4T1^ApoA1^ cells (Fig. 3o), along with increased transcription of downstream genes such as HMGCR and LDLR (Fig. 3p). Furthermore, transfection of 4T1 cells with an LDLR-overexpression plasmid resulted in a slight increase in KRT14 expression (Fig. 3q). The involvement of the ABCA1 receptor in ApoA1-mediated cholesterol efflux was further supported by the presence of the ABCA1 antagonist disulfonic acid hydrate disodium salt (DIDS), which mitigated the downregulation of KRT14 in 4T1^ApoA1^ cells (Fig. 3r). Similarly, the knockdown of ABCA1 expression using shRNA reversed the expression of KRT14 in ApoA1-overexpressing 4T1 cells (Fig. 3s). Consistent with these findings, KRT14 was upregulated in 4T1^APOA1^ cells when cultivated in a medium supplemented with additional cholesterol (Fig. 3t). The restoration of KRT14 expression in 4T1^ApoA1^ cells using KRT14-overexpressing lentivirus increased the number of lung metastatic nodules in mouse models established with these cells (Fig. 3u, v). Moreover, enhanced cell migration, invasive abilities, and clonogenic capabilities were observed in 4T1^ApoA1^ cells transfected with the KRT14-overexpressing plasmid (Fig. 3w-y). Taken together, these results suggest that ApoA1-mediated cholesterol efflux reduces the lung metastases of TNBC by downregulating KRT14 expression.

### ApoA1 downregulates KRT14 transcription by enhancing the expression of the transcription factor FOXO3a

**Fig. 4:**
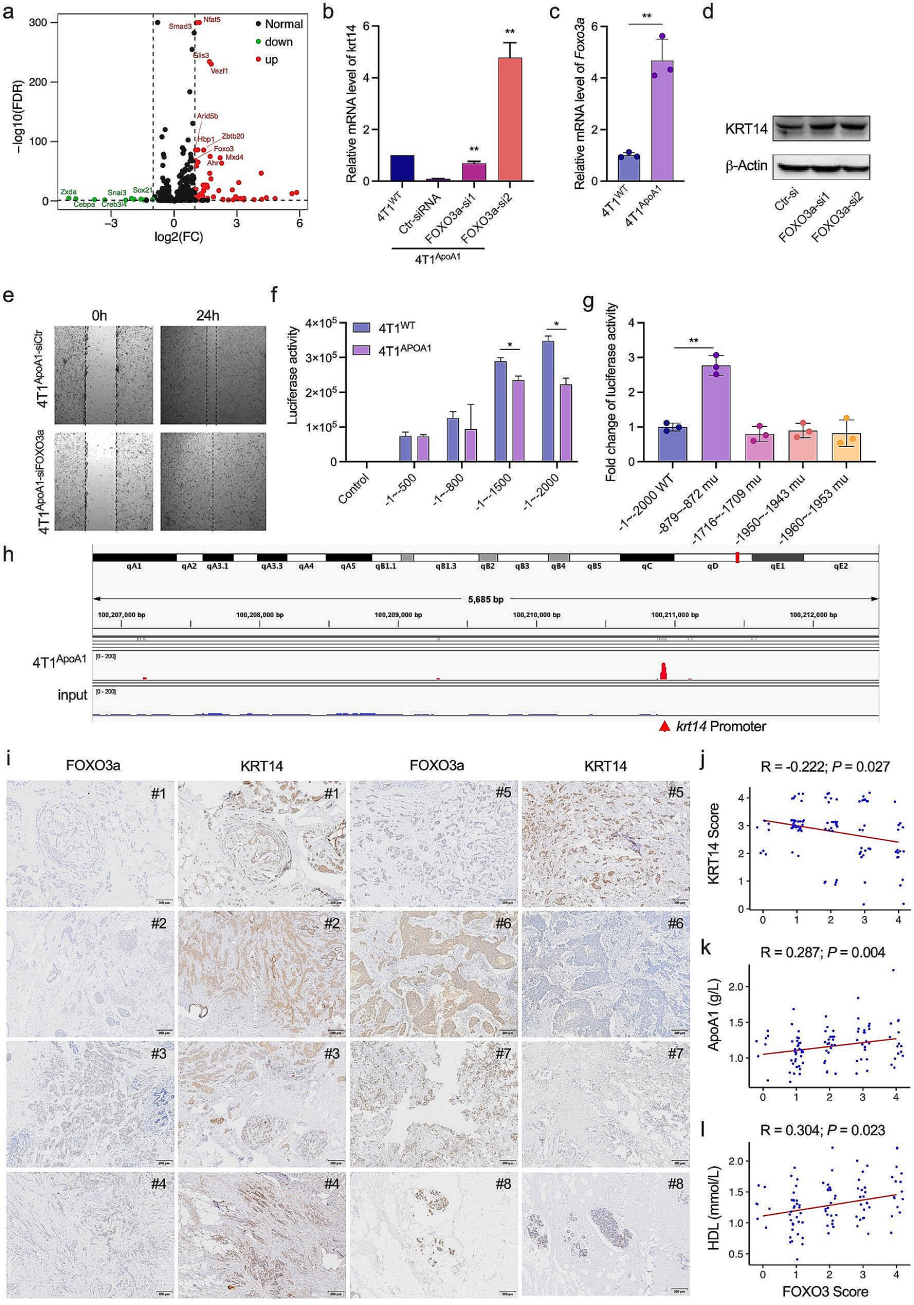
ApoA1 downregulates KRT14 transcription by enhancing the expression of the transcription factor FOXO3a. (a) Volcano plots comparing transcription factors between 4T1^ApoA1^ and 4T1^WT^ cells. (b) 4T1^ApoA1^ cells were transfected with the indicated siRNAs for 48 h. mRNA levels of KRT14 were determined by qPCR. (c) mRNA levels of FOXO3a in 4T1^ApoA1^ and 4T1^WT^ cells determined by qPCR assay. (d) Protein levels of KRT14 determined by western blot. (e) Migration of 4T1 cells transfected with FOXO3a siRNA or control siRNA, determined by wound healing assays. (f) Luciferase reporter gene plasmids controlled by truncated promoters of KRT14 were generated. 4T1^WT^ and 4T1^ApoA1^ cells were transfected with these plasmids, and luciferase activity was measured 48 h later. The lengths of the upstream region of the KRT14 transcription initiation site are indicated (-1~-2000, -1~-1500, -1~-800, and − 1~-500). (g) Luciferase reporter gene plasmids controlled by mutant promoters of KRT14 were generated, and luciferase activity was measured 48 h later. The wide-type promoter of KRT14 is represented by -1~-2000 WT, while the mutation sites are indicated as -879 ~ -872, -1716 ~ -1709, -1950 ~ -1943, and − 1960 ~ -1953. (h) CHIP-seq was performed in 4T1^ApoA1^ lysates using an anti-FOXO3a antibody. IGV analysis of CHIP-seq coverage is shown, indicating the promoter region of KRT14 with a red arrow. (i) Representative image of immunohistochemical staining of KRT14 and FOXO3a in surgical human breast cancer specimens. (j) Correlation analysis of FOXO3a expression with KRT14 expression. k) Correlation analysis of FOXO3a expression with serum ApoA1 levels in patients with breast cancer. l) Correlation analysis of FOXO3a expression with serum HDL levels in patients with breast cancer. *p < 0.05; **p < 0.01

Transcriptional regulation of gene expression often involves the action of transcription factors. To identify potential transcription factors involved in the downregulation of KRT14 by ApoA1, we analyzed transcriptome data from 4T1^ApoA1^ and 4T1^WT^ cells (Fig. 4a) and performed an intersection of the gene list showing differential expression between these twe cell lines, and the predicted gene list of suppressive transcription factors. As shown in Fig. S3a, four genes, including FOXO3a, hypoxia-inducible factor-1 (HIF-1), SMAD Family Member 3 (SMAD3), and Aryl Hydrocarbon Receptor (Ahr), were identified. We observed that the downregulation of KRT14 by ApoA1 was partly abolished in 4T1^ApoA1^ cells transfected with HIF-1, SMAD3 or Ahr siRNA (Fig. S3b). Notably, the ApoA1-induced decrease in KRT14 mRNA levels was increased 5-fold in one FOXO3a-silenced 4T1^ApoA1^ cell line (Fig. 4b). Furthermore, we investigated other transcription factors known to regulate KRT14 expression based on existing literature, such as CCAAT Enhancer Binding Protein α and β (CEBPA and CEBPB), TP53, and SP1. None of them were found to be responsible for the downregulation of KRT14 by ApoA1 (Fig. S3b). Additionally, we observed a significant increase in FOXO3a mRNA level in 4T1^ApoA1^ cells compared to 4T1^WT^ cells, which was confirmed using qRT-PCR assay (Fig. 4c). Additionally, knockdown of FOXO3a using siRNA in 4T1^ApoA1^ cells led to an increase in the expression of KRT14 at the protein level (Fig. 4d) and improve cell migration (Fig. 4e).

To determine the specific DNA sequence in the KRT14 promoter recognized by FOXO3a, we constructed a series of truncated KRT14 promoter-luciferase reporter vectors. Our results indicated that the region located − 800 to − 2000 base pairs (bp) upstream of the transcriptional initiation site was essential for the inhibition of KRT14 transcription (Fig. 4f). Within this region, four FOXO3a binding motifs were expected in the KRT14 promoter. Luciferase assays revealed that mutation of the binding site at -872 to -879 attenuated the suppressive effect of FOXO3a on KRT14 transcription (Fig. 4g). Furthermore, chromatin immunoprecipitation using an antiFOXO3a antibody confirmed the direct binding of FOXO3a to the KRT14 promoter (Fig. 4h). Finally, we analyzed expression levels of KRT14 and FOXO3a in 100 tumor tissues from patients with biopsy-proven breast cancer using immunohistochemistry (Fig. 4i). We observed an inverse correlation between the expression of KRT14 and FOXO3a (Fig. 4j). Moreover, FOXO3a exhibited a positive correlation with serum levels of ApoA1 and HDL (Fig. 4k, l). Overall, these findings suggested that ApoA1 enhances the expression of FOXO3a, which in turn represses KRT14 transcription by directly binding to the KRT14 promoter.

### ApoA1 downregulates KRT14 transcription by promoting the nuclear translocation of FOXO3a

**Fig. 5:**
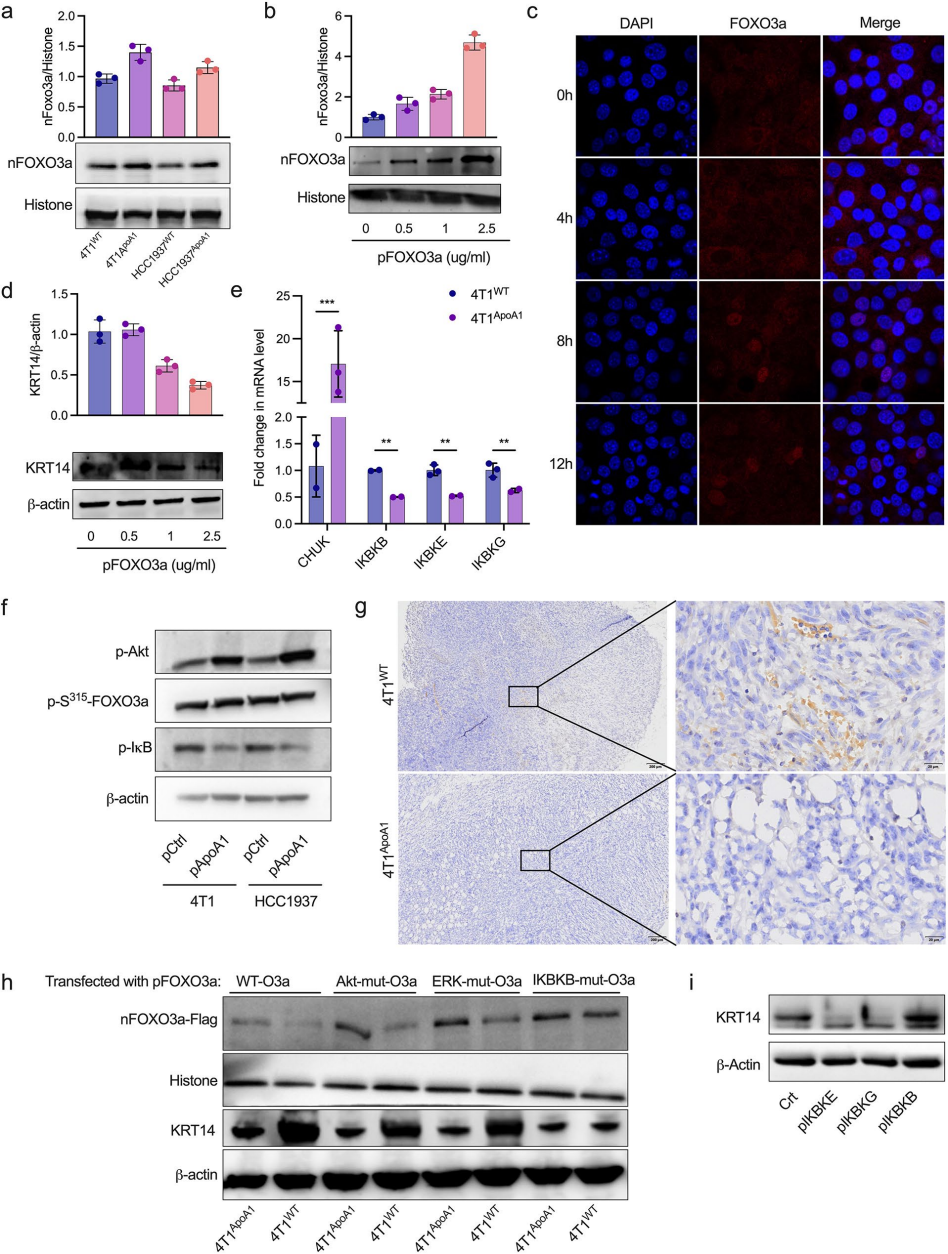
ApoA1 downregulates KRT14 transcription by promoting the nuclear translocation of FOXO3a. (a) The level of nuclear FOXO3a (nFOXO3a) in ApoA1 stable-expressed cells (4T1^ApoA1^ and HCC1937^ApoA1^) was assayed using western blotting. (b) The level of nFOXO3a in 4T1^ApoA1^ cells transfected with the indicated dose of FOXO3a-overexpressing plasmid (pFOXO3a). (c) The nuclear translocation of FOXO3a was detected using immunofluorescence staining. 4T1 cells were transfected with pApoA1 for the indicated time, then fixed and subjected to immunofluorescence staining as described in the Methods section. (d) The expression of KRT14 in 4T1 cells transfected with the indicated dose of pFOXO3a. (e) The difference in mRNA levels of CHUK, IKBKB, IKBKE, and IKBKG between 4T1^ApoA1^ and 4T1^WT^ cells. CHUK: component of inhibitor of nuclear factor Kappa B kinase complex; IKBKB: inhibitor of nuclear factor Kappa B kinase B (IKB) subunit beta; IKBKE: inhibitor of IKB subunit epsilon; IKBKG: inhibitor of IKB subunit gamma. (f) The expression of pAkt, p-S315-FOXO3a (S315 site phosphorylated by Akt), and phosphorylated IκB (p-IκB) in 4T1 and HCC1937 cells transfected with pApoA1 was detected by western blotting. (g) Tumor samples were collected from mice orthotopically inoculated with 4T1^WT^ or 4T1^ApoA1^ cells, and phosphorylated p65 was measured with IHC. (h) The level of nFOXO3a and KRT14 in 4T1^WT^ and 4T1^ApoA1^ cells transfected with a series of plasmids encoding flag-conjugated wild-type FOXO3a (WT) or mutant FOXO3a, which mutate the sites phosphorylated by Akt, ERK, and IKK (Akt-mut-O3a, ERK-mut-O3a, and IKK-mut-O3a). (i) The expression of KRT14 in 4T1^ApoA1^ cells transfected with IKBKB, IKBKE, or IKBKG encoding plasmids. **p < 0.01, ***p < 0.001

Previous studies have shown that FOXO3a can be phosphorylated at distinct sites and sequestered in the cytoplasm [36]. Therefore, we investigated whether ApoA1 influenced the phosphorylation and nuclear translocation of FOXO3a. As depicted in Fig. 5a, increased nuclear levels of FOXO3a (nFOXO3a) were observed in 4T1^ApoA1^ and HCC1937^ApoA1^ cells compared to 4T1^WT^ and HCC1937^WT^ cells. Furthermore, in 4T1^ApoA1^ cells that were transfected with a FOXO3a-overexpressing plasmid, we observed a dose- and time-dependent increase in the expression of nFOXO3a (Fig. 5b). Immunofluorescence staining demonstrated that the FOXO3a was translocated from cytoplasm to nuclear in 4T1 cells that transfected with ApoA1-overexpressing plasmid for 8 h (Fig. 5c). Correspondingly, a dose-dependent decrease in KRT14 expression was observed in 4T1^ApoA1^ cells transfected with FOXO3a-overexpressing plasmid (Fig. 5d).

Previous studies have demonstrated that inhibition of IKBKB (IKKβ, IKK2) leads to nuclear translocation of FOXO3a, and Ser644 of FOXO3a is required for the IKBKB-mediated phosphorylation [37]. To investigate whether ApoA1 affects the expression of IKKs, we performed a qRT-PCR assay and found a significant decrease in the mRNA expression of IKK complex components, including IKBKB, IKBKE (IKK3), and IKBKG, in 4T1^ApoA1^ cells compare to 4T1^WT^ cells (Fig. 5e). Moreover, transfection of an ApoA1-overexpressing plasmid in 4T1 and HCC1937 cells resulted in an increase in Akt phosphorylation and a decrease in IκB phosphorylation (p-IκB). Interestingly, the phosphorylation of FOXO3a at the S315 site (p-S315-FOXO3a, which is the Akt phosphorylation site) was not changed between ApoA1-overexpressing plasmid transfected cells and the control cells (Fig. 5f). Consistently, we also observed a decrease in phosphorylated p65 in tumor tissues from mice inoculated with 4T1^ApoA1^, compared to those inoculated with 4T1^WT^ (Fig. 5g).

To identify the specific kinase responsible for phosphorylating FOXO3a in the current experimental setting, we generated a series plasmids encoding wild-type FOXO3a and FOXO3a mutants. These FOXO3a mutants have mutations at specific sites that render them resistant to phosphorylation modification by Akt, ERK, or IKBKB. As shown in Fig. 5h, only the IKBKB-FOXO3a mutant resulted in a increase of nFOXO3a and decrease in KRT14, indicating that the nuclear translocation of FOXO3a in 4T1 cells primarily relied on the IKBKB pathway. Moreover, overexpression of IKBKB, but not IKBKE, and IKBKG, significantly upregulated the expression of KRT14 in 4T1^ApoA1^ cells (Fig. 5i). These results suggested that the phosphorylation of FOXO3a induced by IKBKB was involved in the ApoA1-mediated regulation of KRT14 in TNBC.

### Oncolytic adenovirus encoding ApoA1 substantially inhibited the growth and metastasis of TNBC

**Fig. 6:**
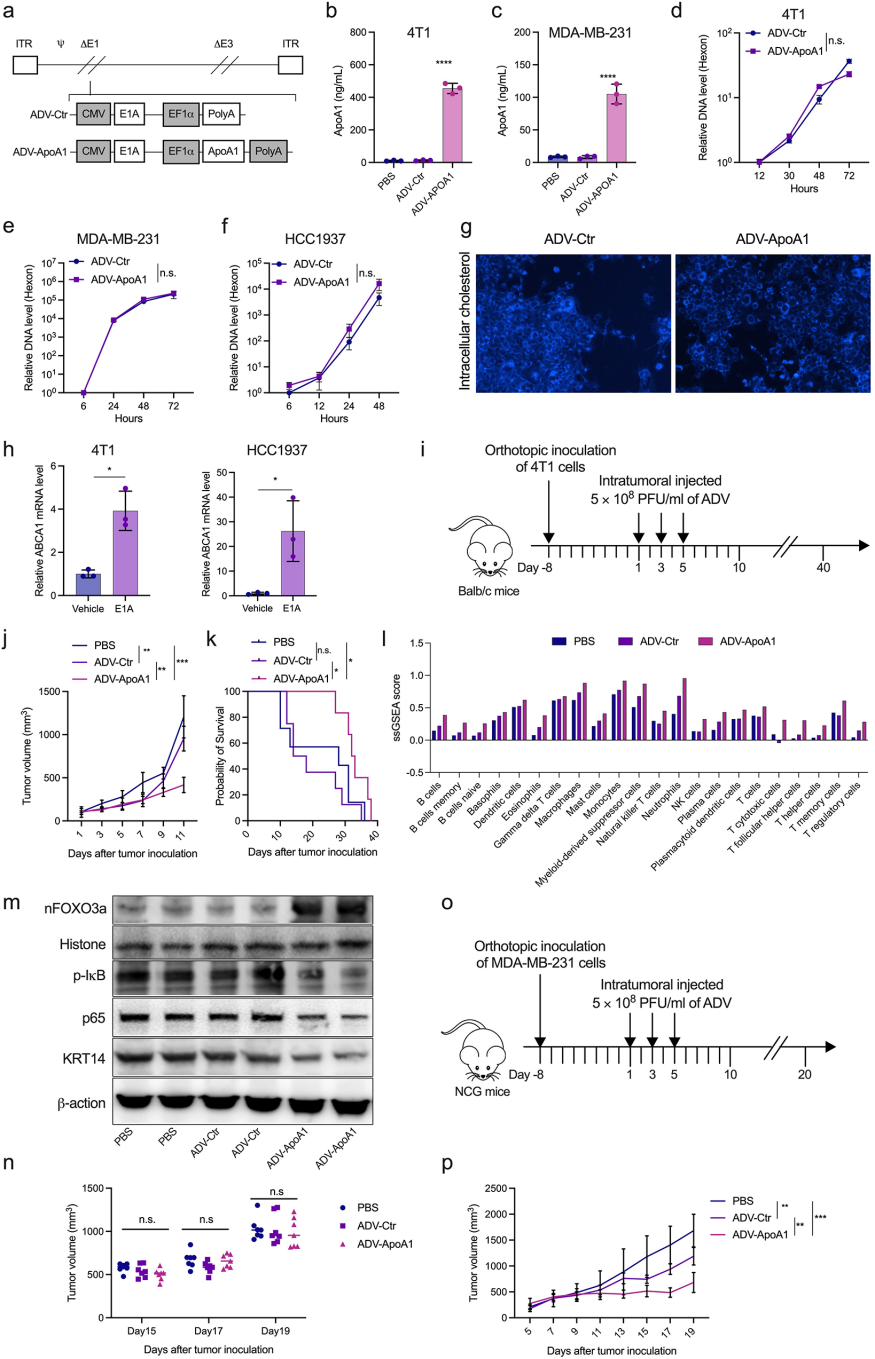
ADV-ApoA1 inhibits TNBC growth in mouse models. (a) Scheme of the adenovirus backbone for ADV-ApoA1 and ADV-Ctrl. (b) and (c) Expression of ApoA1 in the supernatant of culture media from 4T1 and MDA-MB-231 cells infected with ADV-ApoA1 and ADV-Ctrl detected using ELISA. (d) to f) Quantification of the replication ability of ADV-ApoA1 and ADV-Ctrl in 4T1, MDA-MB-231, and HCC1937 cells using qPCR. g) 4T1 cells infected with ADV-ApoA1 or ADV-Ctrl for 48 h, and intracellular cholesterol stained with filipin. h) 4T1 and HCC1937 cells transfected with E1A encoding plasmid in vitro, and mRNA levels of ABCA1 determined by qPCR. i) Treatment scheme of the 4T1 orthotopic breast cancer model. j) and k) Tumor volume curve and survival of mice treated with oncolytic virus. l) Estimation of immune cell infiltration using the ssGSEA method. m) Expression of nFOXO3a, p-IκB, p65, and KRT14 in 4T1 orthotopic tumors treated with PBS, ADV-Ctr, or ADV-ApoA1. n) Tumor volume of nude mice with an orthotopic MDA-MB-231 breast cancer model, which was established and treated similar to 4T1 orthotopic breast cancer model. o) Treatment scheme of the humanized orthotopic MDA-MB-231 breast cancer model in NCG mice. p) Tumor volume curve of mice treated with oncolytic virus. n.s.: no significant difference; *p < 0.05, **p < 0.01, ***p < 0.001, ****p < 0.0001

Considering the inhibitory effect of ApoA1 on lung metastasis in TNBC, we postulated that ApoA1 could be utilized in oncolytic virus therapies. To explore this possibility, we engineered an oncolytic adenovirus encoding ApoA1 (ADV-ApoA1) and assessed its antitumor efficacy (Fig. 6a). Following infection with ADV-ApoA1, 4T1 and MDA-MB-231 cells efficiently expressed and secreted ApoA1 protein (Fig. 6b, c). Furthermore, ADV-ApoA1 effectively replicated in 4T1, MDA-MB-231, and HCC1937 cells, exhibiting similar viral replication capacity to the control Virus (ADV-Ctr) (Fig. 6d-f). Notably, ADV-ApoA1 infection led to a significant reduction in intracellular cholesterol in 4T1 cells, when compared to ADV-Ctrl (Fig. 6g). Additionally, the overexpression of adenovirus early region 1 (E1A) in 4T1 and HCC1937 cells significantly upregulated the expression of ABCA1 (Fig. 6h), indicating a potential synergistic effect of ADV and ApoA1 on cholesterol efflux.

In an orthotopic 4T1 model (Fig. 6i), treatment with ADV-ApoA1 significantly suppressed tumor growth and prolonged the survival of tumor-bearing mice, as compared to ADV-Ctr and PBS (Fig. 6j, k). To investigate the impact of oncolytic viruses on the tumor immune microenvironment, we performed next-generation sequencing on orthotopically transplanted tumors that were intratumorally injected with oncolytic viruses. We then utilized the Single-sample Gene Set Enrichment Analysis (ssGSEA) method to conduct enrichment analysis of gene sets for immune cells. The results depicted in Fig. 6l demonstrate that, compared to the PBS and ADV-Ctr treated groups, the ADV-ApoA1 treated group exhibited a higher presence of immune cells in the tumor microenvironment. Furthermore, ADV-ApoA1 treatment of mice resulted in an increase in nFOXO3a and a decrease in p-IκB, p65, and KRT14 in orthotopic tumors (Fig. 6m). To assess the anti-tumor effects of immune cells associated with oncolytic viruses, we established an orthotopic MDA-MB-231 nude mouse breast cancer model and treated it with intratumoral injections of PBS, ADV-Ctr, and ADV-ApoA1, respectively. As illustrated in Fig. 6n, no differences in tumor volume were observed among the three treatment groups. However, treatment with ADV-ApoA1 significantly reduced tumor growth in an MDA-MB-231 humanized murine TNBC model (Fig. 6o, p).

**Fig. 7:**
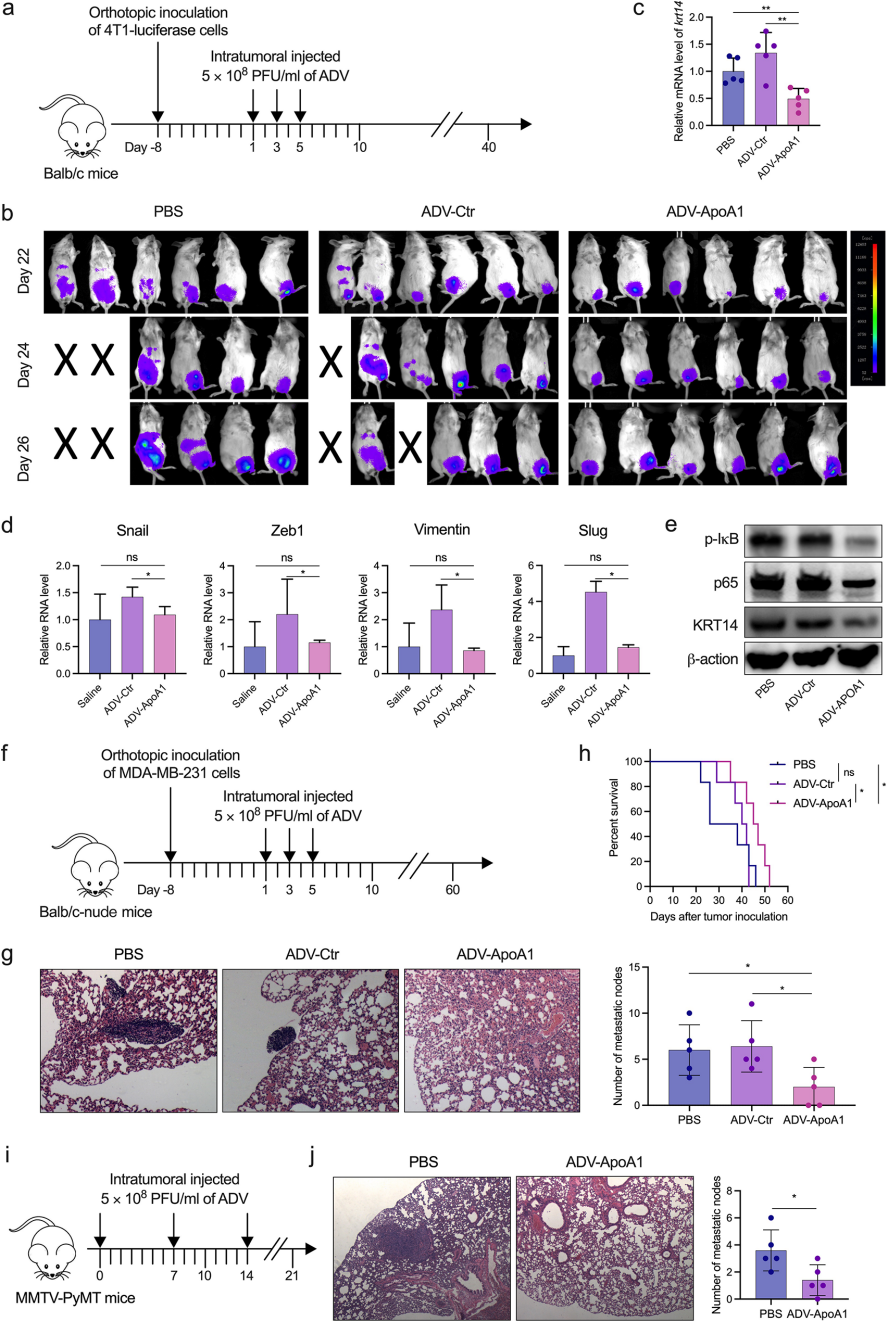
ADV-ApoA1 inhibits TNBC metastasis in mouse models. (a) Treatment scheme of the 4T1-luciferase orthotopic breast cancer model. (b) Monitoring of tumor metastasis using bioluminescence analysis. (c) mRNA level of KRT14 in tissue from the 4T1-luciferase orthotopic breast cancer model, assayed by qPCR. (d) mRNA levels of KRT14 and EMT-associated genes in tissue from the 4T1-luciferase orthotopic breast cancer model, assayed by qPCR. (e) Expression of p-IκB, p65, and KRT14 in 4T1 metastatic tumors of mice that was treated with PBS, ADV-Ctr, or ADV-ApoA1 in orthotopic tumors. (f) Treatment scheme of the MDA-MB-231 orthotopic breast cancer model in nude mice. (g) Mice sacrificed on day 20 after the first oncolytic virus treatment, lung tissue collected and used for H&E staining. The number of metastatic nodes was calculated. (h) Survival curve of mice treated with oncolytic virus. (i) Treatment scheme of the MMTV-PyMT mice. (j) Mice sacrificed three weeks after the first virus injection, lung tissue sections were collected and used for H&E staining. The number of metastatic nodes was calculated. ns: no significant difference; *p < 0.05; **p < 0.01

Subsequently, we established several metastasis models of TNBC and to investigate the anti-tumor effect of ADV-ApoA1 on metastasis. In a luciferase-labeled 4T1 orthotopic model (Fig. 7a), none of the 6 mice treated with ADV-ApoA1 exhibited lung metastases. In contrast, 3 out of 6 mice in the ADV-Ctr or PBS treated groups developed lung metastases, and 2 of them succumbed to lung metastases (Fig. 7b). Notably, treatment with ADV-ApoA1 markedly diminished the transcription of KRT14 in tumor tissues (Fig. 7c). Surprisingly, ADV-Ctr upregulated the expression of EMT-related genes, including snail, vimentin, slug, and zeb1 compare to PBS, whereas ADV-ApoA1 significantly decreased them (Fig. 7d). Consistant with the orthotopic tumor, ADV-ApoA1 treatment of mice resulted in a decrease in p-IκB, p65, and KRT14 in the metastasis c tumors (Fig. 7e). In an MDA-MB-231 orthotopic model (Fig. 7f), treatment with ADV-ApoA1 significantly reduced the number of lung metastatic nodules and extended the survival of tumor-bearing mice (Fig. 7g, h). Similarly, ADV-ApoA1 treatment resulted in a significant reduction in the number of lung metastatic nodules in MMTV-PyMT mice with spontaneous breast cancer (Fig. 7i, j). Taken together, these results suggest that oncolytic virus ADV-ApoA1 effectively inhibits tumor growth, reduces lung metastases, and prolongs the survival of mice with TNBC.

### ADV-ApoA1 demonstrates high safety in Syrian hamsters and Rhesus monkeys

To evaluate the preclinical safety of ADV-ApoA1 in species other than mice, we conducted safety assessments in Syrian hamsters and rhesus monkeys. Animals treated with ADV-ApoA1 did not exhibit any significant changes in food consumption or weight loss compared to the vehicle control group (data not shown). Only mildly elevated levels of ALT (125.0 ± 16.0 U/L vs. 33.6 ± 13.6 U/L prior to viral injection) were observed in rhesus monkeys receiving an intravenous dose of ADV-ApoA1 at 1 × 10^13^ VP/kg (Tables 1 and 2). Histological examination in Rhesus monkeys showed that subcutaneous injection of high-dose ADV-ApoA1 (1.5 × 10^12^ VP/kg) for 6 times did not produce toxic side effects on the heart, spleen, stomach, rectum and testicles, except for causing slight necrosis and immune cell infiltration in the liver (Fig. S4). However, these liver injuries resolved spontaneously after a 30-day recovery period. These data suggest that ADV-ApoA1 is a safe agent in the preclinical setting in treating TNBC.

**Table 1:** Serum biochemistry profiles of Syrian hamsters after ADV-ApoA1 injection

		Control	5 × 10^12^ VP/Kg (DRF-I.H.)	1.2 × 10^12^ VP/Kg (DRF-I.V.)
Male (*n* = 5)	ALT (U/L)	72.2 ± 18.0	43.8 ± 9.7	49.6 ± 8.0
	AST (U/L)	37.2 ± 4.8	34.6 ± 4.3	51.6 ± 13.2
	ALP (U/L)	189.8 ± 8.2	232.0 ± 18.4	199.2 ± 20.1
	CK (U/L)	818.4 ± 301.5	545.6 ± 239.7	789.4 ± 392.3
	LDH (U/L)	145.6 ± 21.0	142.0 ± 26.9	193.0 ± 46.7
	AMY (U/L)	1771.7 ± 242.7	2277.3 ± 260.4	1840.4 ± 144.1
	LIP (U/L)	54.9 ± 5.1	37.0 ± 8.2	50.7 ± 7.6
	BUN (mmol/L)	6.4 ± 1.1	5.7 ± 0.9	5.3 ± 0.5
	CREA (mmol/L)	16.2 ± 3.4	14.0 ± 1.0	15.2 ± 0.8
	GLU (mmol/L)	12.5 ± 1.7	9.3 ± 1.0	10.2 ± 2.3
Female (*n* = 5)	ALT (U/L)	52.8 ± 7.1	35.4 ± 4.8	65.6 ± 23.7
	AST (U/L)	40.8 ± 4.5	43.4 ± 4.8	59.4 ± 11.3
	ALP (U/L)	201.2 ± 18.9	245.2 ± 22.4	201.4 ± 14.9
	CK (U/L)	1005.4 ± 503.0	871.2 ± 458.9	1153.4 ± 395.6
	LDH (U/L)	131.8 ± 31.1	149.6 ± 30.3	200.2 ± 47.5
	AMY (U/L)	2487.7 ± 405.5	2551.1 ± 199.9	2375.0 ± 517.0
	LIP (U/L)	54.5 ± 9.4	45.0 ± 7.0	45.7 ± 6.2
	BUN (mmol/L)	7.0 ± 0.8	5.2 ± 0.8	6.4 ± 0.5
	CREA (mmol/L)	18.8 ± 2.6	14.2 ± 3.6	16.2 ± 2.8
	GLU (mmol/L)	10.3 ± 1.7	8.9 ± 2.0	8.8 ± 2.5

I.H., hypodermic injection; I.V., intravenous injection. DRF, dose range finding. ALT, alanine amiotransferase; AST, aspartate aminotransferase; ALP, alkaline phosphatase; CK, creatine kinase; LDH, lactate dehydrogenase; AMY, serum amylase; LIP, lipase; BUN, blood urea nitrogen; CREA, creatinine; GLU, glucose

**Table 2:** Serum biochemistry profiles of adult Rhesus monkeys after ADV-ApoA1 injection

	Control male (*n* = 6) female (*n* = 6)	5 × 10^12^ VP/Kg (DRF-I.H.) male (*n* = 1) female (*n* = 1)	2.4 × 10^11^ VP/Kg (DRF-I.V.) male (*n* = 1) female (*n* = 1)	1 × 10^13^ VP/Kg (MTD-I.H.) male (*n* = 1) female (*n* = 1)	1 × 10^13^ VP/Kg (MTD-I.V.) male (*n* = 1) female (*n* = 1)
ALT (U/L)	33.6 ± 13.6	31.0 ± 23.0	19.0 ± 5.0	32.0 ± 2.0	125.0 ± 16.0^*^
AST (U/L)	50.1 ± 20.1	50.0 ± 5.0	33.5 ± 0.5	41.5 ± 5.5	70.0 ± 26.0
ALP (U/L)	647.2 ± 214.7	424.0 ± 81.0	803.0 ± 60.0	575.0 ± 14.0	468.5 ± 5.5
CK (U/L)	1036.7 ± 1776.3	1267.0 ± 1112.0	219.0 ± 96.0	452.0 ± 175.0	244.5 ± 104.5
LDH (U/L)	597.9 ± 257.9	689.0 ± 54.0	541.5 ± 33.5	512.0 ± 201.0	571.5 ± 180.5
AMY (U/L)	610.5 ± 145.0	689.2 ± 97.2	615.0 ± 111.9	419.7 ± 22.0	532.6 ± 44.9
LIP (U/L)	27.4 ± 22.0	54.0 ± 38.4	16.5 ± 3.7	18.1 ± 3.8	30.2 ± 3.7
BUN (mmol/L)	7.3 ± 1.7	6.9 ± 1.2	6.3 ± 2.0	6.3 ± 0.1	7.6 ± 1.4
CREA (mmol/L)	46.5 ± 6.9	46.0 ± 3.0	44.0 ± 5.0	47.5 ± 7.5	51.0 ± 4.0
GLU (mmol/L)	4.9 ± 0.6	4.7 ± 0.4	4.6 ± 0.3	4.1 ± 0.2	4.8 ± 0.2

I.H., hypodermic injection; I.V., intravenous injection. DRF, dose range finding (12-Day course of 10 viral injections); MTD, maximum tolerated dose (1 viral injection). ALT, alanine amiotransferase; AST, aspartate aminotransferase; ALP, alkaline phosphatase; CK, creatine kinase; LDH, lactate dehydrogenase; AMY, serum amylase; LIP, lipase; BUN, blood urea nitrogen; CREA, creatinine; GLU, glucose. ^*^P < 0.05, compared with that in untreated group

In summary, our results reveal a novel metastatic regulating axis, cholesterol-IKBKB/FOXO3a/KRT14, through which cholesterol promotes metastasis of TNBC. Manipulating cholesterol metabolism through the oncolytic virus ADV-ApoA1 proves to be an effective and safe treatment strategy for TNBC.

## Discussion

**Fig. 8:**
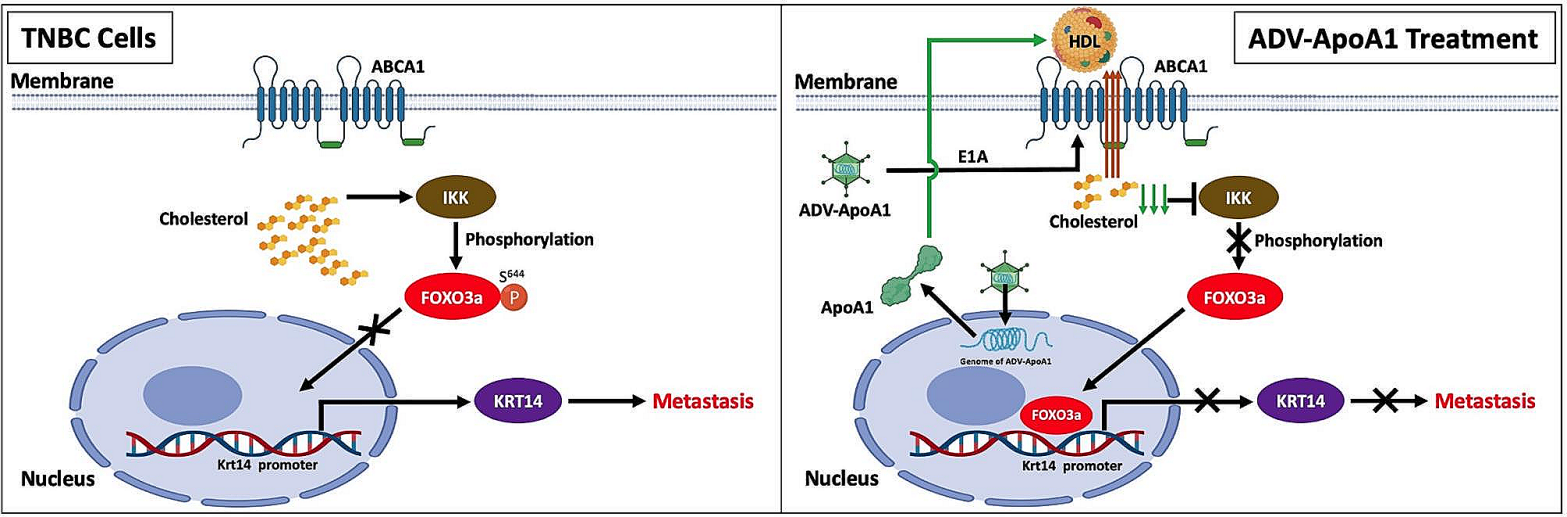
A schematic model visualizing of cholesterol-IKBKB/FOXO3a/KRT14 axis in promoting metastasis of TNBC and the mechanism of ADV-ApoA1 in inhibiting metastasis of TNBC. In cholesterol-IKBKB/FOXO3a/KRT14 axis, KRT14 acts as a critical regulator of TNBC invasion and metastasis, while FOXO3a functions as a transcription repressor of KRT14. ApoA1-mediated cholesterol efflux effectively reduces the phosphorylation of FOXO3a by IKBKB, which leads to the nuclear translocation of FOXO3a and consequently suppresses KRT14 transcription and lung metastasis in TNBC. Replicative ADV-ApoA1 with E1A protein upregulates ABCA1, facilitating ApoA1-mediated cholesterol efflux

Triple-negative breast cancer (TNBC), characterized by its highly aggressive nature, poses significant challenges in terms of treatment [38]. In this study, we elucidated the roles and mechanisms of cholesterol in the progression and metastasis of TNBC. Notably, we identified, for the first time, the inhibitory effect of ApoA1 on TNBC metastasis, achieved through the regulation of the cholesterol/IKK/FOXO3a/KRT14 axis. Furthermore, we developed an oncolytic adenovirus expressing ApoA1, which effectively enhanced cholesterol efflux, resulting in the inhibition of TNBC growth and metastases (Fig. 8). Importantly, this therapeutic approach demonstrated good tolerability in both rhesus monkeys and Syrian hamsters. This study provides insights into the development of effective treatments for TNBC.

KRT14 is a structural constituent of the cytoskeleton and participates in the assembly of desmosomes and hemidesmosomes as a major intermediate filament. KRT14 is considered a regulator of cell-cell and cell-extracellular matrix interactions. Breast cancer cells can propagate KRT14-positive (KRT14^+^) and KRT14-negative (KRT14^−^) daughter cells via the asymmetric division and thereby generate heterogeneity [39]. Compare to KRT14^−^ breast cancer cells, KRT14^+^ breast cancer cells exhibited more invasiveness in three-dimensional organoid assays and an enhanced ability to develop circulating tumor cell clusters. A previous study showed that KRT14^+^ breast cancer cells located at the invasion front or interface between the tumoroid and extracellular collagen and mediated collective dissemination [35, 40]. Another recently published study has demonstrated that EZH2 is capable of catalyzing the trimethylation of lysine 27 of histone H3 (H3K27me3), thereby facilitating the upregulation of KRT14 and promoting peritoneal metastasis in TNBC [41]. Consistent with earlier studies, our findings in this study revealed that knocking down KRT14 led to reduced metastasis of 4T1 breast cancer and prolonged survival in mice. Conversely, we observed that feeding mice a high-cholesterol diet significantly upregulated KRT14 expression and promoted breast cancer metastasis. With these results, we unveiled the relationship between cholesterol and KRT14 in TNBC metastasis, providing a plausible explanation for the previously observed phenomenon of cholesterol promoting TNBC metastasis [42]. Based on this significant finding, we believe that KRT14 represents a promising therapeutic target for TNBC, and interventions aimed at targeting KRT14 may prove to be an effective strategy against TNBC.

Since KRT14 is downstream of cholesterol, it can be regulated by modulating upstream cholesterol metabolism, thereby influencing the metastatic characteristics of breast cancer. There are two approaches to reducing intracellular cholesterol levels: inhibiting *de novo* synthesis and promoting its efflux. Statins have been reported to effectively inhibit de novo cholesterol synthesis and tumor progression [17, 43]. On the other hand, LXRa inhibitors or ApoA1 can be employed to promote cholesterol efflux. However, previous studies have shown that while LXRa inhibitors enhance cholesterol efflux from cells, they may also induce the upregulation of multidrug resistance pumps, leading to chemotherapy resistance [44]. ApoA1, as the primary protein component of HDL, has demonstrated the ability to facilitate cholesterol efflux and reverse transport [34]. Consistent with these reports, our study demonstrates that ApoA1 significantly enhances cholesterol efflux in breast cancer cells, directly reducing intracellular cholesterol levels. Additionally, ApoA1 can lower serum cholesterol levels by increasing serum HDL levels. Notably, we discovered that ApoA1 regulates KRT14 expression through the cholesterol/IKK/FOXO3a axis. Previous studies have confirmed that FOXO3a functions as a tumor suppressor gene in breast cancer, inhibiting VEGF-A/NRP1 signaling and breast cancer metastasis by driving miRNA signatures [45]. In this study, we reveal, for the first time, that FOXO3a directly interacts with the KRT14 promoter, transcriptionally repressing KRT14 expression in breast cancer. Analysis of tumor tissues from breast cancer patients further confirmed the negative correlation between KRT14 and FOXO3a. Previous studies have indicated that FOXO3a is primarily regulated by the PI3K/Akt, ERK, and IKK signaling pathways, and the phosphorylation of FOXO3a affects its nuclear translocation [37, 42, 46]. Our study demonstrates that cholesterol-mediated cytoplasmic retention of FOXO3a primarily occurs via the IKBKB signaling pathway, rather than the PI3K/Akt or ERK signaling pathways, confirming earlier findings that IKK negatively regulates FOXO3a in breast cancer [37].

In our in vitro experiments, we observed that transfecting breast cancer cells with a plasmid overexpressing ApoA1 or infecting them with a lentivirus carrying ApoA1 gene resulted in inhibited migration of breast cancer. Similarly, in mouse models of breast cancer, ApoA1 demonstrated its ability to suppress metastasis of breast cancer. These findings suggest that ApoA1-based gene therapy holds promise as a strategy for treating breast cancer metastasis. Notably, we discovered that E1A, an early protein of adenovirus, significantly upregulates the expression of ABCA1, a membrane-bound receptor essential for ApoA1-mediated cholesterol efflux [47]. This characteristic makes replicative adenovirus an ideal vehicle for delivering ApoA1. Therefore, in this study, we utilized adenovirus carrying ApoA1 to construct a replicative oncolytic adenovirus (ADV-ApoA1) for breast cancer treatment. As anticipated, infection of breast cancer cells with ADV-ApoA1 promoted cholesterol efflux, reduced KRT14 expression, decreased lung metastases in TNBC-bearing mice, and prolonged their survival. To the best of our knowledge, this is the first instance of employing an oncolytic virus to manipulate cholesterol efflux as a means to control TNBC metastasis.

In addition to the cell-intrinsic effects mediated by KRT14, it has been discovered that cholesterol induces CD8^+^ T cell exhaustion in the tumor microenvironment (TME) [48]. Moreover, KRT14 expression has been implicated in MHC class II presentation in breast cancer [35], suggesting that down-regulation of KRT14 in TNBC could result in increased MHC II expression and facilitate the activation of CD4^+^ T cells. In our previous study [49], we discovered that the accumulation of cholesterol in tumor-associated macrophages (TAMs) significantly hinders the mitochondrial translation of TAMs, which ultimately impacts their phagocytic function. Conversely, the intratumoral injection of ADV-ApoA1 can reprogram the lipid metabolism of the GBM tumor microenvironment, modulate cholesterol efflux, restore TAMs’ phagocytosis, and reactivate TAM-T cell anti-tumor immunity. Additionally, the therapeutic efficacy of ADV-ApoA1 in GBM models was nullified after depleting CD8^+^ T cells, indicating that the anti-tumor effect of ADV-ApoA1 is dependent on CD8^+^ T cells. In the current study, we observed that ADV-ApoA1 exhibited a greater capacity to recruit immune cells compared to ADV-Ctr, indicating that ApoA1 may impact the infiltration and activation of immune cells through cholesterol and its downstream signal. Therefore, in immune-competent mice with TNBC, ADV-ApoA1 exhibited therapeutic potential by exerting both cell-intrinsic and cell-extrinsic effects, including the stimulation of antitumor immune responses. To address this question, we utilized nude mice lacking T cells to establish the MDA-MB-231 orthotopic model. In this model, we found that ADV-ApoA1 did not demonstrate superior efficacy compared to ADV-Ctrl or PBS in inhibiting tumor growth. These results indicate that the therapeutic impact of ADV-ApoA1 on tumor growth requires the involvement of T cells, aligning with our previous findings in GBM models. However, in the NCG humanized mouse model (where immune function was restored in immunodeficient mice through intravenous PBMC injection), ADV-ApoA1 exhibited greater efficacy than ADV-Ctrl in impeding tumor growth, further emphasizing the crucial role of T cells in contributing to the anti-tumor effects of ADV-ApoA1. Moreover, even in nude mouse models, ADV-ApoA1 treatment markedly impeded the lung metastasis of breast cancer cells, indicating that the role of ADV-ApoA1 in inhibiting breast cancer metastasis primarily involves the regulation of the cholesterol/IKBKB/FOXO3a/KRT14 axis.

Toxicity and side effects are crucial factors that restrict the clinical application of oncolytic viruses. In this study, we discovered that ADV-ApoA1 exhibited manageable toxicity and did not induce adverse effects in rhesus monkeys and Syrian hamsters, irrespective of whether administered intravenously or intratumorally. These findings provide significant assurance for the future clinical implementation of ADV-ApoA1. In addition, the clinical efficacy and application of adenovirus may be somewhat limited due to the presence of pre-existing neutralizing antibodies in the general population [50]. In this study, ADV-ApoA1 is derived from adenovirus serotype 5, and the neutralizing antibodies induced by it primarily target the adenoviral hexon protein. A promising strategy involving molecular retargeting has been attempted to overcome this challenge. Recently, a study focused on using a combination of mutations in the hexon HVR1 loop with a hexon mutation that abolishes coagulation factor X (FX) binding, as well as a penton mutation that redirects the virus from macrophage β3-containing integrins to integrins with other β subunits present on epithelial tumor cells [51]. In another study, a bifunctional linker containing a tumor-specific ligand and the adenoviral hexon domain DE1 was utilized to conjugate anti-adenoviral antibodies. This approach effectively engages antiviral antibodies to recognize and combat tumors [52]. Moving forward, similar strategies should be explored for ADV-ApoA1 to address the potential impact of neutralizing antibodies on its therapeutic efficacy.

## Conclusions

In this study, we provide evidence that ApoA1 effectively inhibits TNBC metastasis through the regulation of the cholesterol/IKBKB/FOXO3a/KRT14 axis. Furthermore, our findings suggest that the gene therapy strategy utilizing oncolytic adenovirus encoding ApoA1 is not only safe but also holds great promise for TNBC treatment. ADV-ApoA1 emerges as a potent biological agent with significant potential, warranting further investigation in clinical studies.

### Electronic supplementary material

Below is the link to the electronic supplementary material.

Supplementary Material 1

## Data Availability

All data supporting the findings of this study are available within the article and its Supplementary Information.

## Abbreviations

TNBC: Triple-Negative Breast Cancer
KRT14: Keratin 14
ApoA1: Apolipoprotein A1
ADV-ApoA1: Oncolytic Adenovirus Encoding Apoa1
LDL: Low-Density Lipoprotein
HDL: High-Density Lipoprotein
TGs: Triglycerides
Treg: Regulatory T Cells
Th17: T Helper 17 Cells
LDLR: Low Density Lipoprotein Receptor
LXR: Liver X Receptors
IKBKB: Inhibitor Of Nuclear Factor Kappa-B Kinase Subunit Beta
FOXO3a: Forkhead Box Protein O3
ATCC: American Type Culture Collection
DMEM: Dulbecco’s Modified Eagle’s Medium
FBS: Fetal Bovine Serum
MOI: Multiplicity Of Infection
RUF: Fluorescence Intensity
ChIP-Seq: Chromatin Immunoprecipitation Sequencing
qRT-PCR: Quantitative Real-Time Polymerase Chain Reaction
GAPDH: Glyceraldehyde-3-Phosphate Dehydrogenase
siRNA: Small Interfering RNA
PVDF: Polyvinylidene Fluoride
PFA: Paraformaldehyde
EGFP: Enhanced Green Fluorescent Protein
CMV: Cytomegalovirus
EF1a: Elongation Factor 1-Alpha
T2A: Thosea Asigna Virus 2 A
PEI: Polyethyleneimine
TCID50: The Tissue Culture Infectious Dose 50
PBMCs: Peripheral Blood Mononuclear Cells
PFU: A Plaque-Forming Unit
IHC: Immunohistochemistry
HRP: Horseradish Peroxidase
DRF: Dose Range Finding
I.V.: Intravenously
I.H.: Intraperitoneally
CRISPR: Crispr
SAM: Synergistic Activation Mediator
TCGA: The Cancer Genome Atlas
GTEx: The Genotype-Tissue Expression
nTNBC: Non-Triple-Negative Breast Cancer
CT: Computed Tomography
GSEA: Gene Set Enrichment Analysis
HMGCR: 3-Hydroxy-3-Methyl-Glutaryl-Coenzyme A Reductase
SREBP2: Sterol Regulatory Element-Binding Protein 2
DIDS: Disulfonic Acid Hydrate Disodium Salt
ABCA1: Member 1 Of Human Transporter Sub-Family ABCA
nFOXO3a: Nuclear Forkhead Box Protein O3
IKK: Inhibitor Of Nuclear Factor Kappa-B Kinase
IKBKG: Inhibitor Of Nuclear Factor Kappa-B Kinase Subunit Gamma
IKBKE: Inhibitor Of Nuclear Factor Kappa-B Kinase Subunit Epsilon
Akt: AKT Serine Threonine Kinase
ERK: Extracellular Signal-Regulated Kinase
E1A: Early Region 1
ssGSEA: Single-Sample Gene Set Enrichment Analysis
LXRa: Liver X Receptor-Α
MHC: Major Histocompatibility Complex
TME: Tumor Microenvironment
