# Supplementary material for: Oncolytic adenovirus encoding apolipoprotein A1 suppresses metastasis of triple-negative breast cancer in mice
Source: J Exp Clin Cancer Res. 2024 Apr 3;43:102. doi: 10.1186/s13046-024-03011-0 (PMC10988920; doi:10.1186/s13046-024-03011-0)
Supplement: Supplementary file 1 — Supplementary Material 1 [file 13046_2024_3011_MOESM1_ESM.pdf]

Supplementary information

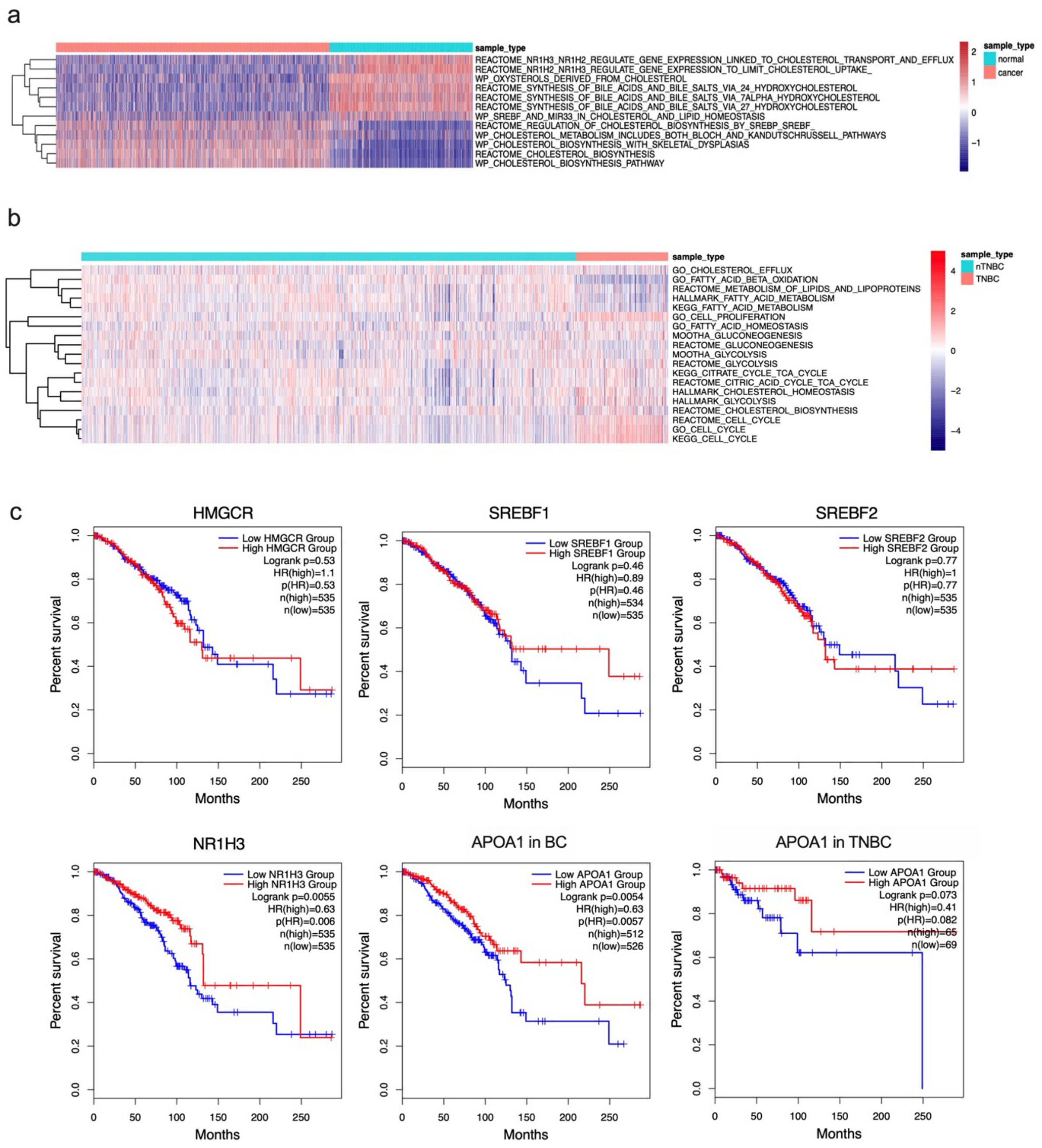

Figure S1, Single-ample gene set enrichment analysis (ssGSEA) of cholesterol metabolism pathways and the association between cholesterol biosynthesis/efflux genes and patient survival in breast cancer patients. a) Enrichment of indicated pathways with data normalized from -2 (Blue) to 2 (Red) in BRCA cancers (n=1092, data from TCGA) and normal breast tissue (n=572, data form TCGA and GTEx). b) Enrichment of indicated pathways with data normalized from -4 (Blue) to 4 (Red) in triple-negative breast cancers (TNBC, n=299) and non-triple-negative breast cancers (nTNBC, n=1605). The gene expression data were obtained from cBioPortal. c) Correlation between cholesterol biosynthesis/efflux genes and survival of the

breast cancer patients using the Gene Expression Profiling Interactive Analysis (GEPIA) online tool (http://gepia.cancer-pku.cn).

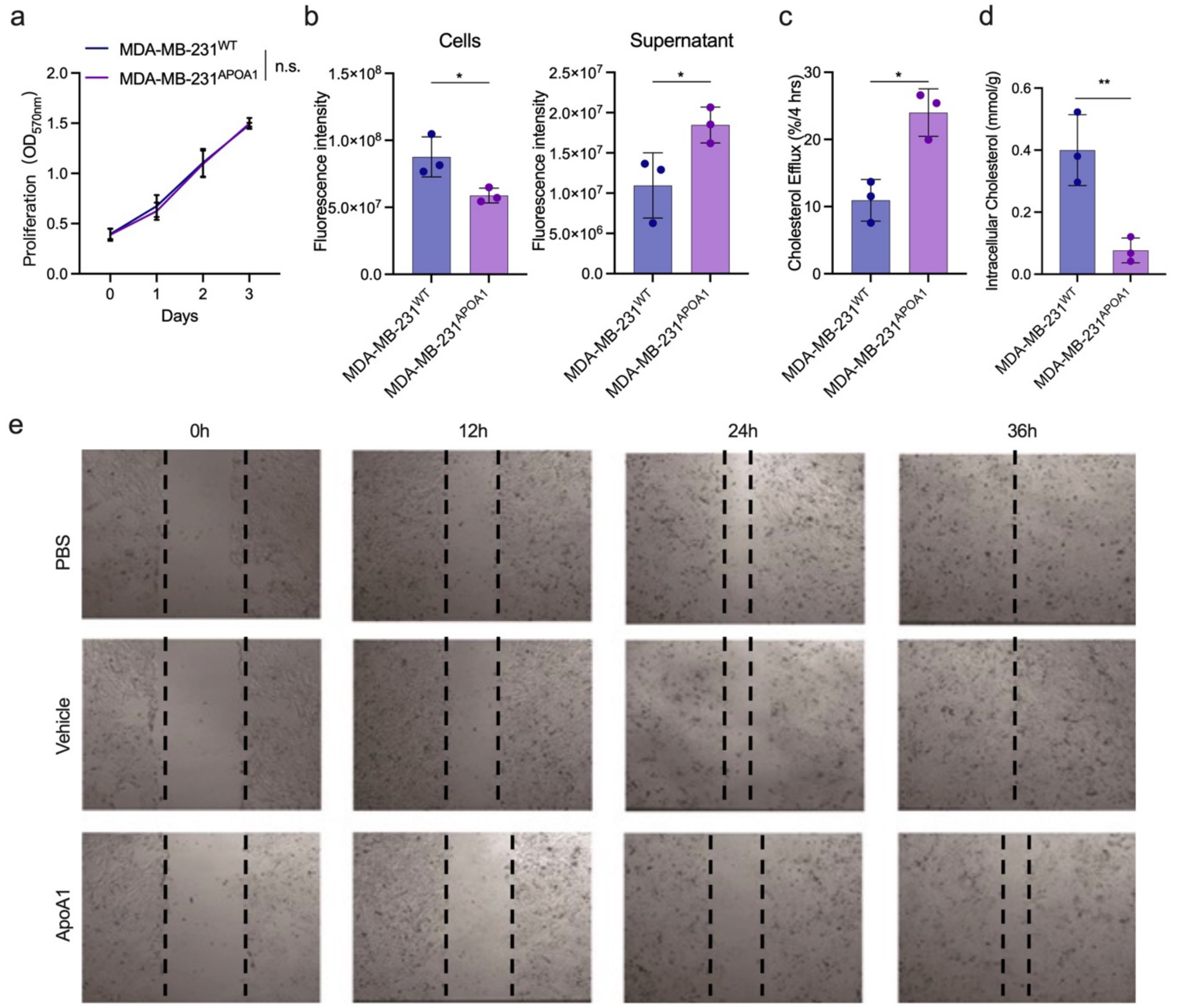

Figure S2 ApoA1 promote cholesterol efflux and inhibits migration in MDA-MB-231 cells. A stable MDA-MB-231 cell line overexpressing ApoA1, MDA-MB-231^{ApoA1}, was generated through lentivirus infection. Proliferation of MDA-MB-231^{ApoA1} and the parental cell line MDA-MB-231^{WT} was determined using MTT assay. b) and c) Cholesterol efflux was measured using a cholesterol efflux assay kit. d) Intracellular cholesterol levels were measured using spectrophotometric tests. e) Migration of MDA-MB-231 cells transfected with either ApoA1 expressing plasmid (pApoA1) or control plasmid (vehicle) was tested using wound healing assays. ns: no significant different; *$p < 0.05$; **$p < 0.01$.

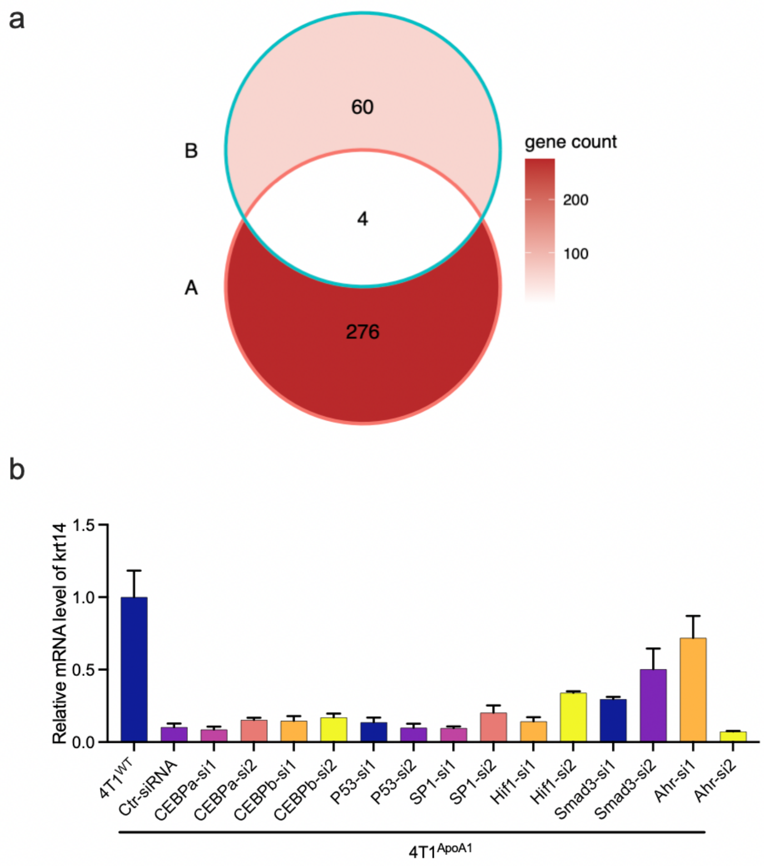

Figure S3 Screening for transcription factors involved in ApoA1 down-regulation of KRT14 transcription. a) Venn plot of intersection of two gene lists: one predicting transcription factors that may bind to KRT14 (60), and the other identifying suppressive transcription factors with differing expression between 4T1ApoA1 and 4T1WT (276). Four gene including FOXO3A, hypoxia-inducible factor-1 (HIF-1), SMAD Family Member 3 (SMAD3), and Aryl Hydrocarbon Receptor (Ahr) were screened out. b) Gene knockdown assay was used to determine the transcription factors involved in ApoA1 down-regulation of KRT14 transcription. 4T1ApoA1 cells were transfected with the indicated siRNAs for 48 hours. mRNA levels of KRT14 were determined by a qPCR assay.

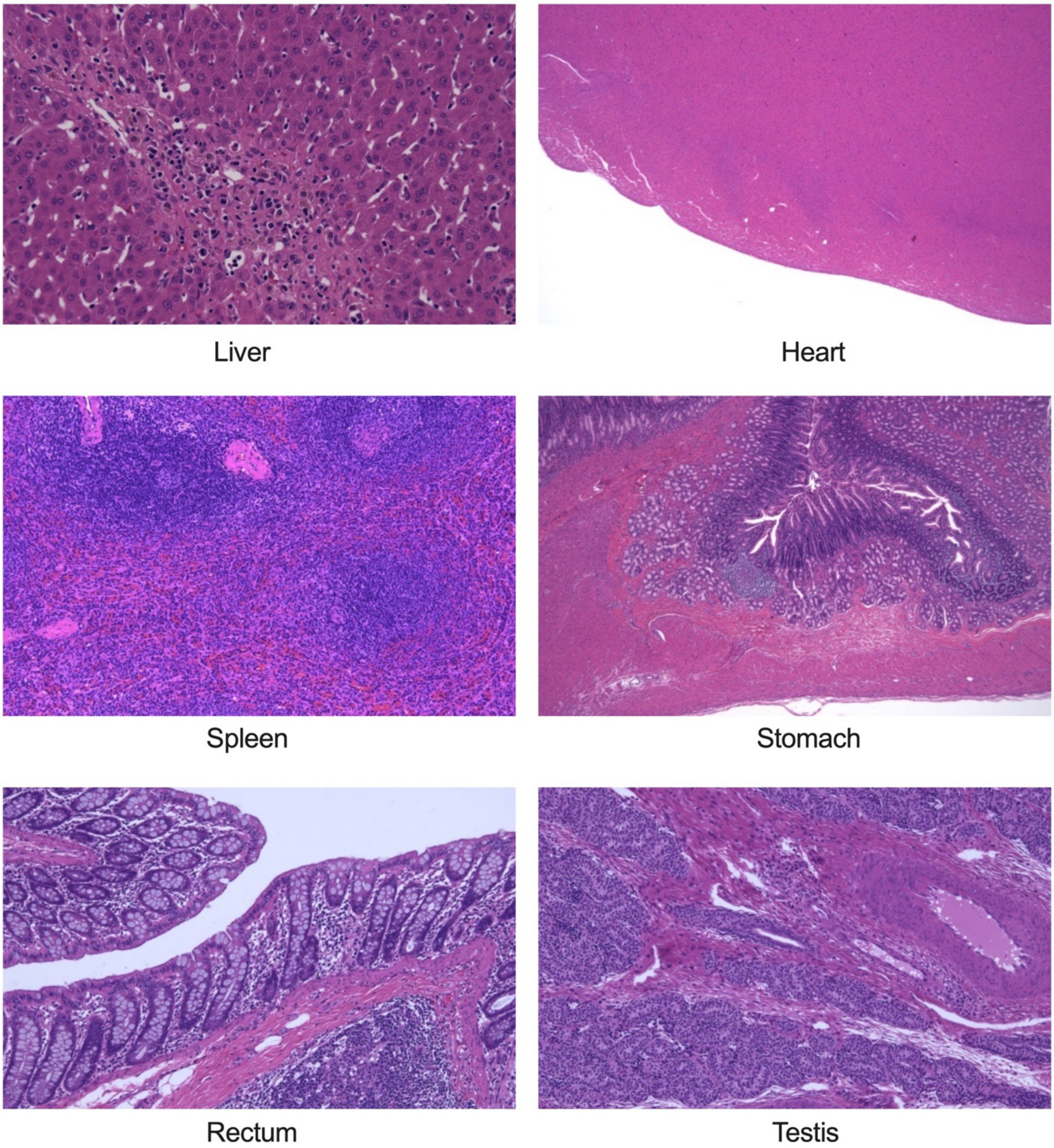

Figure S4 Safety assessment subcutaneous injection of ADV-ApoA1 in Rhesus monkeys. Rhesus monkeys were injected subcutaneously with ADV-ApoA1 (1.5×10^{12} VP/kg) six times. Tissues were collected 24 hours after the last injection for H&E staining to assess toxicity.

Table S1 Primers for quantitative real-time PCR (qPCR).

Gene	5' forward primer	3' reverse primer
Mouse FOXO3a	GAGCTGGAGCTCGAACCTT	TCCTCGGGGATCATGGAGTC
Mouse KRT14	CAGAGCGGCAAGAGTGAGAT	GGAAGATGAAAGGTGGGCGT
Mouse ABCA1	CCTCAGCCATGACCTGCCTTGTAG	CCGAGGAAGACGTGGACACCTTC
Mouse Vimentin	TGAGATCGCCACCTACAGGA	TTGCGCTCCTGAAAAACTGC
Mouse Slug	CATCCTTGGGGCGTGTAAGT	ATGGCATGGGGGTCTGAAAG
Mouse Zeb1	TCCGGCTCCTACTCTCAACA	GCTCCAACCTCCACTGTACC
Mouse SNAIL	ATGGAGTGCCTTTGTACCCG	CAGTAACCACCCTGCTGAGG
Mouse β-actin	GTACCACCATGTACCCAGGC	AACGCAGCTCAGTAACAGTCC
Mouse GAPDH	TCTCCTGCGACTTCAACA	TGTAGCCGTATTCATTGTCA
E1A	CCGAAGATCCCAACGAGGAG	TTGGCATAGAAACCGGACCC
Human ABCA1	GTTAGGAAACCTGCTGCCCT	ATGCCACACACAGGACGTAG
Human β-actin	TGGCACCCAGCACAATGAAG	CTGTCACCTTCACCGTTCCA

Table S2 siRNA for gene knock-down

Gene	Sense strand（5’-3’）	Antisense strand（5’-3’）
FOXO3a-1	GCUCUUGGUGGAUCAUCAATT	UUGAUGAUCCACCAAGAGCTT
FOXO3a-2	GCCAGUCUAUGCAAACCCUTT	AGGGUUUGCAUAGACUGGCTT
Ahr-1	GCAACUAGAGCAACAGCAATT	UUGCUGUUGCUCUAGUUGCTT
Ahr-2	GCAAAUCAACGGCACGUUUTT	AAACGUGCCGUUGAUUUGCTT
Smad3-1	ACGCAGAACGUGAACACCAAGUGCA	UGCACUUGGUGUUCACGUUCUGCGU
Smad3-2	CAUGAAGAAGGAUGAAGUGUGUGUA	UACACACACUUCAUCCUUCUUCAUG
Hlf1-1	CCUUCAUCCUGAAGACGCAUUUAGU	ACUAAAUGCGUCUUCAGGAUGAAGG
Hlf1-2	ACGGAGAUACUUUCCAGCUGGAAUA	UAUUCCAGCUGGAAAGUAUCUCCGU
p53-1	GACCUAUCCUUACCAUCAUTT	AUGAUGGUAAGGAUAGGUCTT
p53-2	CCACUUGAUGGAGAGUAUUTT	AAUACUCUCCAUCAAGUGGTT
Sp1-1	CCAGCAACAUGGGAAUUAUTT	AUAAUUCCCAUGGUUGCUGGTT
Sp1-2	GCGGCAAAGUAUAUGGCAATT	UUGCCAUAUACUUUGCCGCTT
Cebpa-1	GCGCAAGAGCCGAGAUAAATT	UUUAUCUCGGCUCUUGCGCTT
Cebpa-2	GGAGUUGACCAGUGACAAUTT	AUUGUCACUGGUCAACUCCTT
Cebpb-1	AGGCCAAGAUGCGCAACCUTT	AGGUUGCGCAUCUUGGCCUTT
Cebpb-2	GGAACUUGUUCAAGCAGCUTT	AGCUGCUUGAACAAGUUCCTT
ABCA1 -1	GUGUCUACGUGCAACAGAUTT	AUCUGUUGCACGUAGACACTT
ABCA1 -2	CUAGCCUGCUGUCUCCUGUGGC	GCCACAGGAGACAGCAGGCUAG
